# Iron-overload injury and cardiomyopathy in acquired and genetic models is attenuated by resveratrol therapy

**DOI:** 10.1038/srep18132

**Published:** 2015-12-07

**Authors:** Subhash K. Das, Wang Wang, Pavel Zhabyeyev, Ratnadeep Basu, Brent McLean, Dong Fan, Nirmal Parajuli, Jessica DesAulniers, Vaibhav B. Patel, Roger J. Hajjar, Jason R. B. Dyck, Zamaneh Kassiri, Gavin Y. Oudit

**Affiliations:** 1 Division of Cardiology, Department of Medicine, New York; 2 Mazankowski Alberta Heart Institute, New York; 3 Department of Physiology, University of Alberta, New York; 4 Mount Sinai School of Medicine, New York; 5 Departments of Pediatrics and Pharmacology, University of Alberta, Edmonton, Canada

## Abstract

Iron-overload cardiomyopathy is a prevalent cause of heart failure on a world-wide basis and is a major cause of mortality and morbidity in patients with secondary iron-overload and genetic hemochromatosis. We investigated the therapeutic effects of resveratrol in acquired and genetic models of iron-overload cardiomyopathy. Murine iron-overload models showed cardiac iron-overload, increased oxidative stress, altered Ca^2+^ homeostasis and myocardial fibrosis resulting in heart disease. Iron-overload increased nuclear and acetylated levels of FOXO1 with corresponding inverse changes in SIRT1 levels in the heart corrected by resveratrol therapy. Resveratrol, reduced the pathological remodeling and improved cardiac function in murine models of acquired and genetic iron-overload at varying stages of iron-overload. Echocardiography and hemodynamic analysis revealed a complete normalization of iron-overload mediated diastolic and systolic dysfunction in response to resveratrol therapy. Myocardial SERCA2a levels were reduced in iron-overloaded hearts and resveratrol therapy restored SERCA2a levels and corrected altered Ca^2+^ homeostasis. Iron-mediated pro-oxidant and pro-fibrotic effects in human and murine cardiomyocytes and cardiofibroblasts were suppressed by resveratrol which correlated with reduction in iron-induced myocardial oxidative stress and myocardial fibrosis. Resveratrol represents a clinically and economically feasible therapeutic intervention to reduce the global burden from iron-overload cardiomyopathy at early and chronic stages of iron-overload.

Iron-overload is driven by hemochromatosis and secondary iron-overload conditions^1^,^2^,^3^,^4^,^5^,^6^. Thalassemia, sickle cell anemia and hemochromatosis are among the most frequently inherited disorders world-wide^3^,^7^. The prevalence and global clinical burden of iron-overload is increasing with epidemic proportions but therapy remains limited^5^,^7^,^8^. Iron-overload cardiomyopathy is the most common cause of mortality in patients with secondary iron-overload, and is a major co-morbidity in patients with genetic hemochromatosis^5^,^6^,^9^,^10^,^11^,^12^,^13^. Altered iron homeostasis allows uncontrolled iron entry and deposition in different organs including the heart leading to progressive tissue damage and end-organ failure^14^,^15^. Excess entry of iron leads to transferrin saturation and non-transferrin bound iron (NTBI) accumulation in iron-overload conditions^15^,^16^,^17^. Iron-induced oxidative stress plays a fundamental role in the pathogenesis of iron-overload mediated heart disease^16^,^18^,^19^. The formation of labile NTBI alters the pro-oxidant/antioxidant balance leading to a pro-oxidant state with increased free radical production, oxidative stress and cellular damage^18^,^20^,^21^. Current antioxidants are ineffective because of failure to target the correct intracellular compartment of reactive oxygen species in the setting of iron-overload and some anti-oxidants such as ascorbic acid can be readily converted into a free radical pro-oxidant^22^,^23^.

The basic molecular mechanism of iron-overload cardiomyopathy has not been elucidated and strategies to treat this global epidemic are limited. Iron-overload in humans leads to an advanced cardiomyopathy^5^,^6^,^9^,^12^, and the development and validation of pre-clinical models of iron-overload cardiomyopathy are important for the discovery of new therapies^16^,^24^,^25^. We identified the SIRT1/FOXO1 axis as a key pathway involved in iron-overload. Resveratrol (RSV) is a natural polyphenolic flavonoid with a unique ability to activate SIRT1 and has key pleotropic and anti-oxidant properties^26^,^27^,^28^,^29^,^30^. We used dietary supplementation with RSV to rescue the heart disease in murine models of secondary iron-overload and genetic hemochromatosis. We also demonstrated that iron-mediated pathological effects on human cardiomyocytes and cardiofibroblasts were prevented by RSV. Collectively, our results strongly suggest that RSV is a useful therapy to reduce the global burden of iron-overload cardiomyopathy.

## Results

### A key role of SIRT1/FOXO1 pathway in iron-overload induced myocardial injury

**Figure 1:**
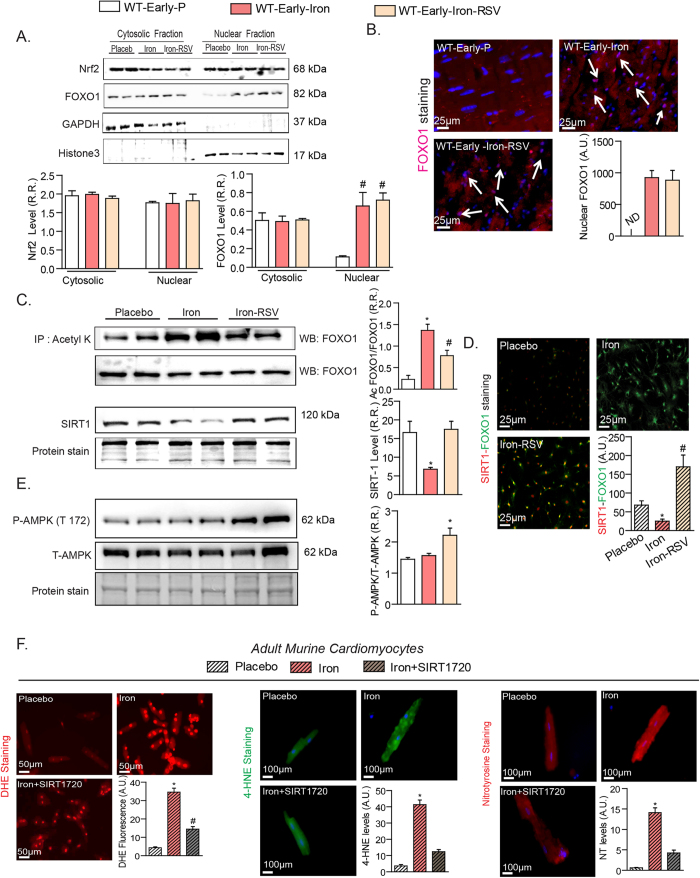
Iron-overload alters myocardial SIRT1/FOXO1 signaling which is restored by RSV. (**A,B**) Western blot analysis and quantification of two major transcriptional factors, Nrf2 and FOXO1, showing no change in Nrf2 levels but increased nuclear levels of FOXO1 in early iron-overloaded hearts (**A**) with immunofluorescence staining in myocardial tissue with early iron-overload confirming increased nuclear FOXO1 levels as illustrated by the white arrows (**B**). (**C,D**) Immunoprecipitated cardiac acetylated FOXO1 increased in response to iron-overload which was markedly suppressed by resveratrol (RSV) with corresponding inverse changes in SIRT1 levels (**C**) while immunofluorescence staining for FOXO1 (green) and SIRT1 (red) in cultured and stretched murine LV cardiofibroblasts showing that in response iron exposure nuclear FOXO1 increased with reduced SIRT1 levels, while RSV (100 μM) prevents the loss of SIRT1 without affecting the increased total FOXO1 levels (**D**). Resveratrol therapy increased the phosphorylation of AMPK (threonine-172) in iron-overloaded myocardium (**E**). SIRT1 activator, SRT1720 (1 μM), prevents iron-induced oxidative stress in cardiomyocytes based on dihydroethidium (DHE) staining for superoxide levels, 4-hydroxynonenal (4-HNE) and nitrotyrosine immunofluorescence (**F**). R.R. = relative ratio; A.U. = arbitrary unit. n = 3 repeats from n = 2 hearts. *p < 0.05 compared with all other groups; ^#^p < 0.05 compared with the placebo group.

We investigated the molecular basis of iron-induced myocardial injury and focused on the SIRT1/FOXO-1 pathway and the modulation by RSV therapy in early iron-overloaded WT mice. Forkhead box-O (FoxOs) and Nrf2 transcription factors transduce a wide range of extracellular signals, while FOXO1 is regulated by SIRT1^31^. While Nrf2 levels were unchanged, total nuclear and acetylated FOXO1 levels increased in response to iron-overload which was markedly suppressed by RSV with corresponding inverse changes in SIRT1 levels (Fig. 1A–C). Immunofluorescence staining in cultured and stretched cardiofibroblasts exposed to iron showed reduced SIRT1 levels which was restored by RSV and co-localized with FOXO1 (Fig. 1D). Resveratrol therapy also increased phosphorylation of AMPK, a key mediator of its beneficial action^32^,^33^, in iron-overloaded myocardium (Fig. 1E). We next used a specific and potent SIRT1 activator, SRT1720^27^,^34^, to critically examine the role of the SIRT1 pathway in iron-mediated injury. Isolated adult murine cardiomyocytes showed a strong pro-oxidant response to exposure to iron based on superoxide (dihydroethidium, DHE), aldehyde (4-hydroxynonenal, 4-HNE) and nitrotyrosine levels which were markedly suppressed by SRT1720 (Fig. 1F). These results provide instrumental evidence for a critical role of the SIRT1-FOXO1 axis in iron-mediated myocardial injury and in mediating RSV protective effects in iron-overload cardiomyopathy.

### Downregulation of Sarcoendoplasmic reticulum Calcium ATPase2a (SERCA2a) in early iron-overload cardiomyopathy: impact of SERCA2a gene and RSV therapies

**Figure 2:**
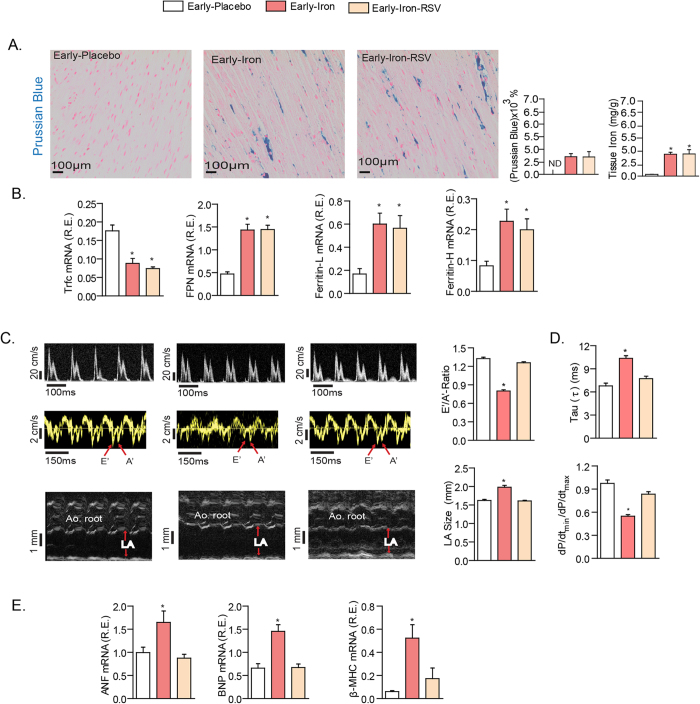
Pathological myocardial remodeling and diastolic dysfunction in early iron-overload is completely rescued by RSV therapy independent of myocardial iron-deposition. Prussian blue staining and quantification of iron deposition in early iron-overloaded mice showing myocardial iron-overload (**A**) and altered expression of iron metabolic genes, transferrin receptor 1 (Trfc1), ferroportin (FPN), and ferritin light (**L**) and heavy (**H**) chain (**B**). Resveratrol did not affect myocardial iron deposition or the expression iron metabolism genes (**A,B**). Echocardiographic assessment of heart function illustrated by transmitral filling pattern (top panel), tissue Doppler (middle panel) and left atrial (LA) size (bottom panel) (**C**) showing adverse remodeling and diastolic dysfunction in early iron-overloaded wildtype mice. Invasive hemodynamic measurement revealed impaired myocardial relaxation as the primary functional abnormality (**D**). Resveratrol (RSV) treatment normalized the diastolic dysfunction (**C,D**) and the expression of myocardial disease markers (**E**). E’ = early tissue Doppler velocity; A’ = tissue Doppler due to atrial contraction; LA = left atrial; Tau = LV relaxation time constant; dP/dt = rate of change in LV pressure; ANF = atrial natriuretic factor; BNP = brain natriuretic peptide; β-MHC = beta-myosin heavy chain. n = 8 for gene expression analysis; n = 6 for placebo and n = 8 for iron-treated groups. ND = not detected; *p < 0.05 compared with the placebo group.

We explored the mechanism of iron-induced heart disease at an early stage of acquired iron-overload in WT mice which displayed clear evidence of iron injury as reflected by myocardial accumulation of iron (Fig. 2A) and the increased and decreased expression of iron metabolic genes, ferritin L/H and ferroportin, and transferrin receptor 1 (Trfc1), respectively (Fig. 2B). The myocardial injury in early iron-overload was associated with diastolic dysfunction driven by impaired myocardial relaxation as shown by echocardiographic assessment using transmitral Doppler flow and tissue Doppler imaging and invasive pressure-volume loop analysis (Fig. 2C,D; Supplemental Tables 2–3) without myocardial fibrosis or myocardial inflammation (Supplemental Figure 1). Resveratrol suppressed expression of myocardial disease markers, atrial natriuretic factor (ANF), brain natriuretic peptide (BNP) and beta myosin heavy chain (β-MHC) (Fig. 2E) in early iron-overload which correlated with correction of iron-overload induced diastolic dysfunction (Fig. 2C,D; Supplemental Tables 2–3) without affecting myocardial iron levels (Fig. 2A,B).

**Figure 3:**
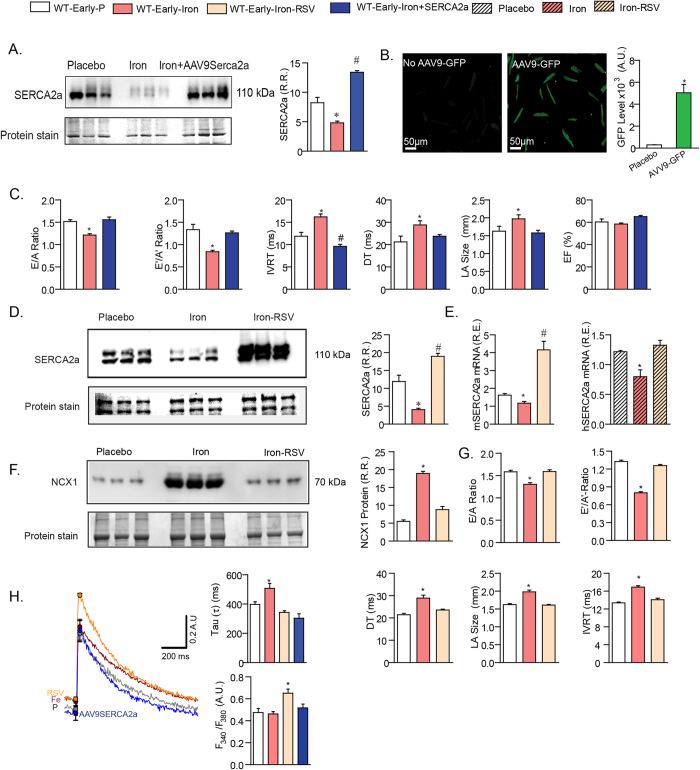
Early iron-overload cardiomyopathy is driven by downregulation of SERCA2a: rescue with adenoviral transfer of SERCA2a and RSV. (**A,B**) Western blot analysis and quantification shows a marked decreased in myocardial SERCA2a level (**A**) which was prevented by *in vivo* adeno-viral gene therapy (AAV9) confirmed in isolated adult ventricular cardiomyocytes following *in vivo* AAV9 delivery of green fluorescent protein (GFP) showing a high yield of efficient gene delivery to the heart (**B**). Assessment of diastolic function using transthoracic echocardiography showing *in vivo* gene delivery of SERCA2 normalized the diastolic dysfunction associated with early iron-overload (**C**). (**D,E**) Western blot analysis revealed a dramatic corrective action of resveratrol (RSV) on the reduced SERCA2a levels (**D**) which correlated with the ability of RSV to prevent iron-induced downregulation of *Serca2a* mRNA expression in mouse (m) and human (h) LV cardiomyocytes (**E**,**F**) Western blot analysis of sodium-calcium exchanger-1 (NCX-1) showing increased levels in early iron-overload which was normalized in response to RSV. (**G**) Functional assessment of heart function showing diastolic dysfunction in early iron-overloaded wildtype mice was completely normalized by RSV therapy. (**H**) Ca^2+^ transients in ventricular cardiomyocytes showing elevated diastolic Ca^2+^ levels and prolongation of Ca^2+^ decay, and correction by SERCA2a gene therapy and RSV. R.R. = relative ratio; R.E. = relative expression; E = early LV transmitral filling velocity; A = LV transmitral filling due to atrial contraction; DT = deceleration time; LA = left atrial; EF = ejection fraction. E’ = early tissue Doppler velocity; A’ = tissue Doppler due to atrial contraction; IVRT = isovolumetric relaxation time. n = 8–12 for functional studies; n = 8 for expression analysis and n = 3–4 for Western blot analysis. *p < 0.05 compared with all other groups; ^#^p < 0.05 compared with the placebo group.

These results identify an abnormality of the cardiomyocytes in early iron-overload which correlated with a significant reduction in myocardial SERCA2a levels (Fig. 3A). Importantly, *in vivo* adenoviral gene delivery of *Serca2a* to mice with iron-overload cardiomyopathy restored SERCA2a levels in the heart (Fig. 3A,B) resulting in complete correction of diastolic dysfunction (Fig. 3C). Interestingly, RSV increased SERCA2a levels (Fig. 3D) which correlated with RSV mediated reversal of iron-induced downregulation of *Serca2a* mRNA in murine and human cardiomyocytes (Fig. 3E). Increased sodium-calcium exchanger level, another key feature of heart disease, was also normalized by RSV therapy (Fig. 3F). Our results highlight a key role of altered Ca^2+^ regulatory proteins in iron-overload cardiomyopathy and therefore we examined whole cell Ca^2+^ transients in isolated cardiomyocytes from early iron-overloaded mice. Importantly, Ca^2+^ transients were prolonged in isolated ventricular iron-overloaded cardiomyocytes and normalized by SERCA2 gene therapy and RSV (Fig. 3H) which correlated with restoration of normal diastolic function and reversal of disease markers. Our results illustrate the key role of abnormal Ca^2+^ cycling driven by reduced SERCA2a levels in iron-overload cardiomyopathy with RSV having a profound corrective effect on SERCA2a levels, Ca^2+^ cycling and cardiac dysfunction.

### Resveratrol suppressed iron-induced increased oxidative stress in cardiomyocytes and in the myocardium of murine iron-overload models

**Figure 4:**
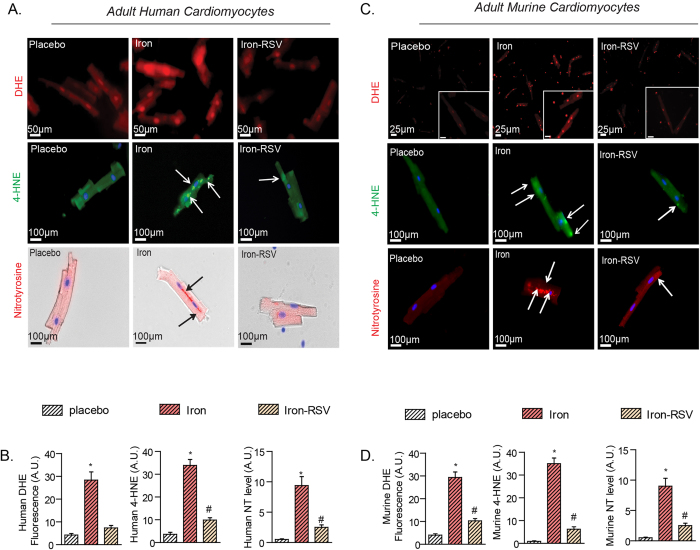
Iron-induced pro-oxidant effects in human and murine cardiomyocytes and in murine models of iron-overload are prevented by RSV. (**A,B**) Isolated adult LV human cardiomyocytes display a pronounced pro-oxidant phenotype after exposure to iron with increased dihydroethidium (DHE) staining for superoxide levels (top), 4-hydroxynonenal (4-HNE) immunofluorescence (middle), nitrotyrosine (NT) immunofluorescence (bottom) (**A**) and quantification of oxidative stress (**B**), while resveratrol (RSV; 100 μM) markedly suppressed iron-induced cellular oxidative stress. (**C,D**) Murine LV cardiomyocytes mirrored similar responses to iron as seen in human LV cardiomyocytes and iron-mediated cellular oxidative stress as illustrated by increased DHE staining, 4-HNE and nitrotyrosine immunofluorescence (**C**) and quantification of oxidative stress (**D**) was markedly suppressed by treatment with RSV. DHE fluorescence and is predominantly nuclear while 4-HNE and nitrotyrosine immunofluorescence are more diffuse and highlighted by the white arrows. n = 4 for immunofluorescence analysis; n = 8 for biochemical and gene expression analysis. *p < 0.05 compared with all other groups; ^#^p < 0.05 compared with the placebo group.

**Figure 5:**
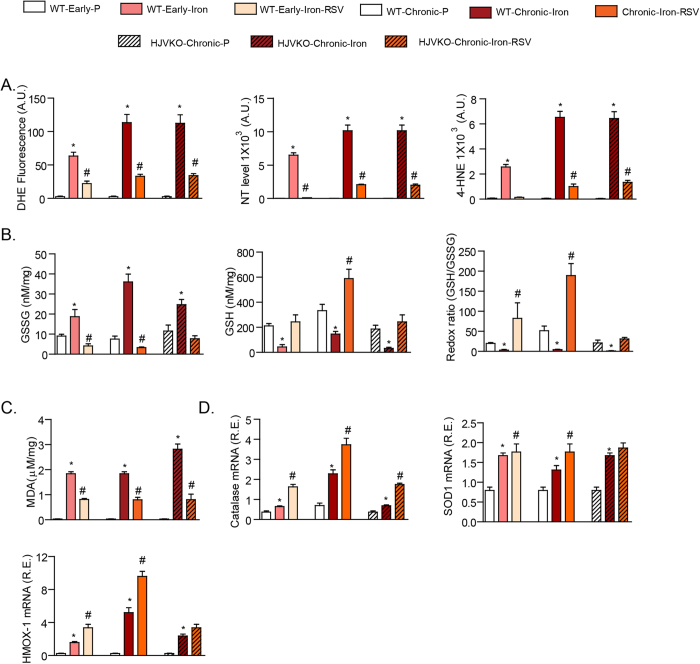
Iron-induced oxidative stress in early and chronic murine models of iron-overload is prevented by RSV. (**A**) Dihydroethidium fluorescence, 4-hydroxynonenal (4-HNE) and nitrotyrosine (NT) immunostaining confirmed increased myocardial oxidative stress in murine models of iron-overload and the therapeutic effects of resveratrol (RSV). (**B**) Myocardial levels of reduced glutathione (GSH), oxidized glutathione (GSSG) and the redox ratio, and the myocardial lipid peroxidation product, malondialdehyde (MDA) (**C**) were altered demonstrating biochemical evidence of increased oxidative damage and reduced anti-oxidant reserve in early and chronic iron-overloaded hearts, markedly corrected by oral RSV therapy. (**D**) Resveratrol potentiated the upregulation of key anti-oxidant enzymes, catalase (CAT), superoxide dismutase 1 (SOD1) and heme oxygenase 1 (HMOX1), in early and chronic iron-overloaded hearts. A.U. = arbitrary unit; R.E. relative expression; LV = left ventricle; n = 8 for biochemical and expression analyses. *p < 0.05 compared with all other groups; ^#^p < 0.05 compared with the placebo group.

Iron-mediated Fenton reaction leads to the formation of free radicals^16^,^18^,^19^. We isolated healthy human left ventricular cardiomyocytes (Supplemental Figure 2) and showed that exposure to iron triggered oxidative stress with increased superoxide (DHE), aldehyde (4-hydroxynonenal, 4-HNE) and nitrotyrosine levels (Fig. 4A,B) which reflect reactive oxygen species, lipid peroxidation and reactive nitrogen species, respectively. Resveratrol completely prevented the iron-mediated oxidative stress on human ventricular cardiomyocytes demonstrating its potent anti-oxidant properties (Fig. 4A,B). We used a similar approach in murine ventricular cardiomyocytes and showed a conserved anti-oxidant response of RSV against iron-mediated oxidative stress (Fig. 4C,D). We next investigated the *in vivo* anti-oxidant effects of RSV in murine models of iron-overload. In addition to early iron-overload, we also used a chronic acquired iron-overload model in WT mice^16^,^18^, and importantly, we generated a chronic genetic hemochromatosis model by aging HJVKO mice fed an iron-enriched diet^24^. Our acquired and genetic murine models of chronic iron-overload clearly demonstrated myocardial iron accumulation and altered expression of genes involved in myocardial iron homeostasis characterized by increased expression of ferritin L/H and ferroportin, and decreased expression of Trfc1 (Supplemental Figure 3). *In vivo* analysis of early and chronic acquired and genetic murine models of iron-overload using DHE fluorescence, 4-HNE and nitrotyrosine immunostaining showed a strong increase in myocardial oxidative stress which was markedly decreased in response to RSV therapy (Fig. 5A; Supplemental Figure 4). Biochemical assessment of myocardial oxidative stress showed increased oxidized glutathione (GSSG) coupled with decreased reduced glutathione (GSH) and redox ratio (Fig. 5B) along with the generation of the lipid peroxidation product, malondialdehyde (MDA) (Fig. 5C), consistent with iron-mediated myocardial oxidative stress. Resveratrol mediated activation of the SIRT1 pathway^26^,^27^,^31^ normalized iron-induced oxidative stress in early and chronic iron-overloaded WT hearts and chronic iron-overloaded HJVKO hearts illustrated by reduced levels of free radicals, lipid peroxidation products and increased GSH levels (Fig. 5A–C; Supplemental Figure 4) which correlated with increased expression of key anti-oxidant genes, catalase, superoxide dismutase 1 and heme oxygenase 1 (Hmox-1; Fig. 5D). These results illustrate a key anti-oxidant effect of RSV against iron-induced oxidative stress at the cellular and myocardial level.

### Resveratrol prevents pro-fibrotic effects in murine and human cardiofibroblasts, and iron-induced myocardial fibrosis and cardiac dysfunction

**Figure 6:**
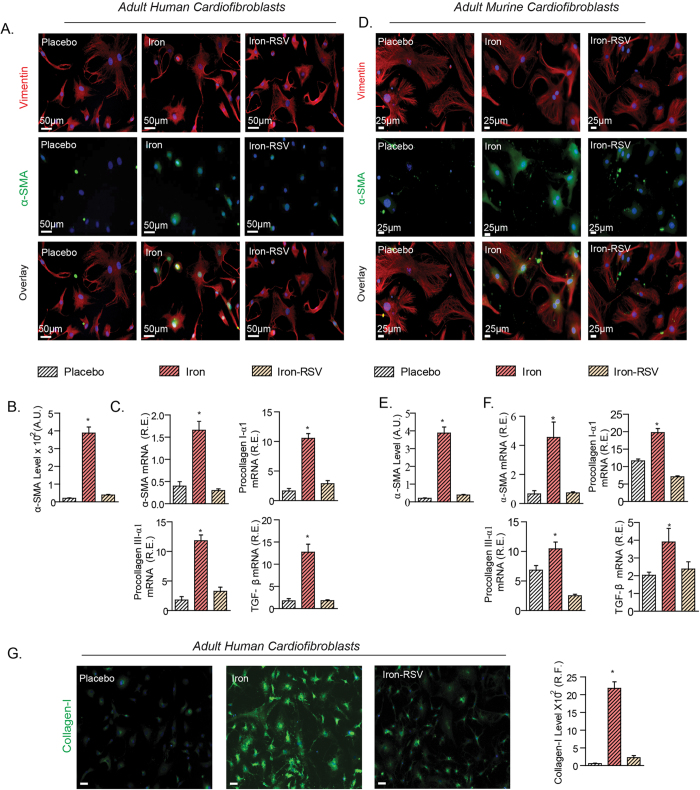
Iron-induced profibrotic effects in human and murine cardiofibroblasts are suppressed by RSV. (**A–C**) Cultured and cyclically stretched adult human LV cardiofibroblasts mounted a pro-fibrotic response to exposure to iron (20 μg/ml) resulting in increased immunostaining for alpha-smooth muscle actin (α-SMA) (**A,B**), and mRNA expression of α-SMA, TGFβ1, pro-collagen type IIIα1, and pro-collagen type Iα1 (**C**) which was prevented by resveratrol (RSV; 100 μM). (**D–F**) Murine LV cardiofibroblasts cultured and cyclically stretched showed a similar pro-fibrotic response when exposed to iron (20 μg/ml) with increased levels of alpha-smooth muscle actin (α-SMA) (**D,E**) and upregulation of the expression of pro-fibrotic genes, pro-collagen Iα1 and IIIα1, α-SMA, TGFβ1, was normalized in response to RSV (100 μM) (**F**). (**G**) Human cardiofibroblasts also showed increased collagen I levels in response to iron (20 μg/ml) which was largely prevented by RSV treatment. A.U. = arbitrary unit; R.E. = relative expression; R.F. = relative fraction; α-SMA = alpha smooth muscle actin; TGFβ1 = transforming growth factor beta1; n = 4 for immunofluorescence analysis; n = 8 for expression analysis. *p < 0.05 compared with all other groups.

We next examined the pro-fibrotic effects of iron and the therapeutic potential of RSV. To enhance the translational impact of our findings, we investigated the impact of iron on human ventricular cardiofibroblasts subjected to cyclical stretching to simulate the cardiac cycle. Exposure to iron transformed human ventricular cardiofibroblasts into an activated myofibroblast phenotype characterize by increased levels of alpha-smooth muscle actin (αSMA) and collagen I, and increased expression of pro-collagen I and III, transforming growth factor beta (TGFβ) and αSMA (Fig. 6A–C). Similarly, in murine ventricular cardiofibroblasts, iron-mediated pro-fibrotic gene expression changes and the increase in collagen I and III levels as also observed in murine ventricular fibroblasts (Fig. 6D–F). Immunohistochemical staining for collagen I confirmed a pro-fibrotic effect of iron in human cardiofibroblasts (Fig. 6G). Resveratrol prevented iron-induced activation of human and murine cardiofibroblasts illustrated by normalization of pro-fibrotic gene expression and αSMA and collagen I levels (Fig. 6A–G). These phenotypic changes in cardiofibroblasts are consistent with the restoration of normal SIRT1 levels coupled with co-localization with FOXO1 in these cells (Fig. 1D).

**Figure 7:**
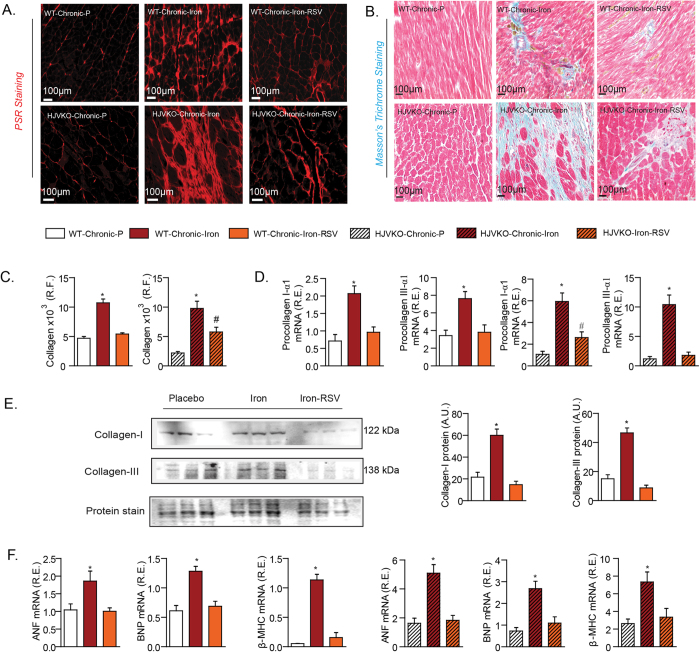
Increased myocardial fibrosis associated with chronic iron-overload cardiomyopathy is completely rescued by RSV therapy. (**A–C**) Histological assessment of myocardial fibrosis using picro-sirius red (PSR) (**A**) and Masson’s trichrome (**B**) staining and quantification of fibrosis (**C**) revealed increased myocardial interstitial and perivascular fibrosis in the chronic iron-overloaded hearts. Expression analysis of myocardial pro-collagen Iα1 and pro-collagen IIIα1 (**D**) and Western blot analysis of myocardial collagen I and collagen III levels (**E**) in chronic iron-overloaded hearts revealed increased levels consistent with a pro-fibrotic state. Resveratrol therapy prevented the increased in myocardial fibrosis based on histological, gene expression and Western blot analysis (**A–E**). Expression analysis of myocardial disease markers in chronic iron-overload models showing a complete normalization of the expression of disease markers in response to resveratrol (RSV) therapy (**F**). A.U. = arbitrary unit; R.E. = relative expression; R.F. = relative fraction; ANF = atrial natriuretic factor; BNP = brain natriuretic peptide; β-MHC = beta-myosin heavy chain. n = 4 for histological analyses; n = 6 for Western blot and n = 8 for expression analyses. *p < 0.05 compared with all other groups; ^#^p < 0.05 compared with the placebo group.

**Figure 8:**
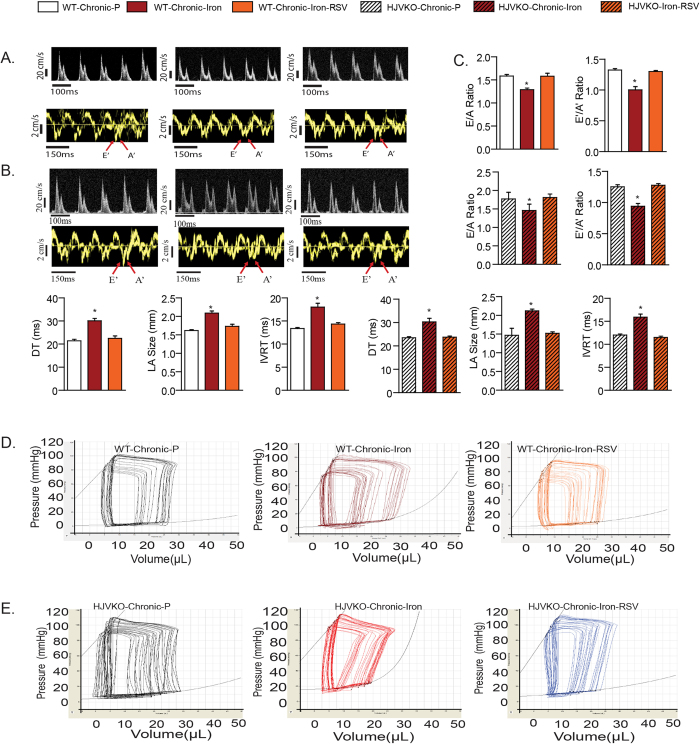
Resveratrol therapy completely rescued the cardiac dysfunction in chronic iron-overloaded wildtype and hemojuvelin knockout mice. Echocardiographic assessment of heart function with transmitral filling pattern (top panel) and tissue Doppler (bottom panel) illustrating diastolic dysfunction in chronic iron-overloaded wildtype mice (**A**) and hemojuvelin knockout (HJVKO) mice (**B**) and quantification (**C**) of the echocardiographic assessment showing diastolic dysfunction. Resveratrol (RSV) treatment completely normalized the diastolic dysfunction in wildtype and HJVKO models of chronic iron-overload (**A–C**). Invasive hemodynamic assessment illustrated by representative pressure-volume tracings confirming load-independent diastolic dysfunction in chronic iron-overloaded wildtype mice (**D**) and HJVKO mice (**E**). E = early LV transmitral filling velocity; A = LV transmitral filling due to atrial contraction; E’ = early tissue Doppler velocity; A’ = tissue Doppler due to atrial contraction; DT = deceleration time; LA = left atrial; IVRT = isovolumetric relaxation time. n = 8 for the placebo groups and n = 10–12 for the iron-treated groups. *p < 0.05 compared with all other groups; ^#^p < 0.05 compared with the placebo group.

Chronic iron-overload *in vivo* resulted in marked increase in myocardial interstitial and perivascular fibrosis (Fig. 7A–C), along with increased pro-collagen I and III mRNA and with increased collagen I and III levels protein levels (Fig. 7D,E) in the absence of myocardial inflammation (Supplemental Figure 5). Resveratrol therapy resulted in marked suppression of myocardial fibrosis *in vivo*, consistent with its *in vitro* anti-fibrotic effects. Interestingly, increased expression of myocardial disease markers, ANF, BNP and β-MHC were also all rescued by RSV treatment in chronic iron-overloaded wildtype and HJVKO hearts (Fig. 7F). These results are consistent with a primary and direct pro-fibrotic effect of iron-overload on cardiofibroblasts as the primary trigger of the increased myocardial fibrosis in chronic iron-overload. Importantly, functional analysis showed that the severe diastolic dysfunction in chronic iron-overload hearts was completely rescued by RSV therapy based on echocardiography (Fig. 8A–C; Supplemental Tables 4–5) and invasive pressure-volume loop hemodynamic analysis (Fig. 8D,E; Supplemental Tables 6–7). In particular, end-diastolic pressure volume relationship, a relatively load-independent index of myocardial stiffness, was markedly increased in response to chronic iron-overload and corrected by RSV therapy (Fig. 8D,E; Supplemental Tables 6–7). Our results demonstrate that chronic iron-overload results in increased myocardial fibrosis as a key driver of heart disease and RSV mediates a pronounced therapeutic effect against iron-induced pro-fibrotic effects.

## Discussion

Therapeutic options for iron-overload cardiomyopathy are limited and there is a clear and urgent need for better therapies to curtail its high degree of mortality and morbidity^5^,^6^,^7^. Our acquired and genetic murine models of iron-overload recapitulate essential features of clinical iron-overload and its associated heart disease. The therapeutic effects of RSV prevented and rescued iron-induced oxidative stress and profibrotic effects in both acquired and genetic models of iron-overload at early and chronic stages of iron-overload cardiomyopathy. Notably, iron-induced oxidative stress in human cardiomyocytes and cardiofibroblasts was completely prevented by RSV directly supporting a possible therapeutic effect in patients with iron-overload. We showed that RSV therapy prevents and rescues the iron-induced pathological events including Ca^2+^ dysregulation, oxidative stress and myocardial fibrosis. Hemojuvelin knockout (HJVKO) mice, a pre-clinical model of juvenile hemochromatosis (type 2 primary hemochromatosis)^3^,^24^, are resistant to iron-induced end-organ pathology^24^,^35^ requiring the use of an iron-enriched diet and aging in order to elucidate significant heart disease. The myocardial iron levels obtained in our murine models (2.5–10 mg/g LV dry weight) are similar to myocardial iron levels (3.5–9.2 mg/g dry LV weight) reported in patients with iron-overload cardiomyopathy and heart failure^14^. In HJVKO mice, RSV therapy was used following 3 months of iron-overload providing further evidence that RSV therapeutic effects are not blunted by pre-existing iron-overload.

We identified the SIRT1/FOXO1 pathway as clearly altered in the heart in response to iron-overload and RSV is a natural polyphenol with a unique ability to activate SIRT1 and has key anti-oxidant properties^26^,^27^,^28^. Deacetylation of FOXOs by SIRT1 protects cellular function during stress conditions and SIRT1 deacetylates FOXO1 and facilitates its nuclear translocation^31^. Resveratrol is a natural polyphenol with antioxidant and metabolic properties due partly to its ability to activate SIRT1^26^,^27^,^29^,^30^,^36^. SIRT1 deacetylates a variety of proteins and regulates genomic integrity, inflammatory responses, mitochondrial function and stress resistance^27^,^28^. Resveratrol therapy resulted in reduced acetylation of myocardial nuclear FOXO1 in response to iron-overload providing a key molecular basis for RSV therapeutic action. The altered redox state of iron-overload coupled with the redox-sensitivity of SIRT1 deacetylase activity likely created a unique environment whereby the therapeutic effects of RSV are enhanced. We used high doses of oral RSV therapy (240–320 mg/kg body weight) in our *in vivo* experiments to ensure adequate bioavailability. Resveratrol used at 300 mg per kg body weight showed no detrimental effects in rats^37^ and a dose of 320 mg per kg was associated with protection from pressure-overload induced heart failure in mice^38^. Given the detrimental effects of iron-overload on mitochondrial function^19^, therapeutic effects of RSV on mitochondria possibly via AMPK activation^32^ may also contribute to its beneficial effects in iron-overload cardiomyopathy. Moreover, our experimental design cannot distinguish between RSV therapeutic effects via SIRT1 modulation versus a direct antioxidant action.

Heart disease is a characteristic feature of iron-overload and is associated with diastolic dysfunction and a late-stage dilated cardiomyopathy^5^,^6^,^16^. Diastolic function depends on two major components, active relaxation and passive stiffness of the myocardium. We showed that early stage of iron-overload induced selective diastolic dysfunction with reduced *Serca2a* mRNA and protein levels leading to abnormal Ca^2+^ cycling. SERCA2a is the dominant mediator of Ca^2+^ re-uptake and reduced SERCA2a function impairs myocardial relaxation leading to diastolic dysfunction^39^,^40^, a phenotype similar to the early iron-overloaded hearts. Importantly, adenoviral gene therapy mediated correction of SERCA2a, normalized the abnormal Ca^2+^ cycling and diastolic dysfunction. Iron-induced suppression of SERCA2 expression in murine and human cardiomyocytes was prevented by RSV resulting in the preservation of SERCA2a protein levels, restoration of normal diastolic function and alleviation of heart disease in early iron-overloaded hearts. Oxidative stress has been linked to decreased SERCA2a activity^41^ with a key role of the SUMOylation pathway^42^,^43^. While both N-acetylcysteine (NAC) and RSV are well-known antioxidants, their mechanisms of action on preserving SERCA2a function are different. N-acetylcysteine prevents oxidative damage to SERCA2a^41^ likely by modulating reduced glutathione levels while we have shown that RSV increased SERCA2a mRNA and protein levels. In contrast to early iron-overload, chronic iron-overloaded hearts displayed marked adverse remodeling of the extracellular matrix with increased interstitial and perivascular fibrosis leading to diastolic dysfunction characterized by increased passive myocardial stiffness. Resveratrol had a marked anti-fibrotic effect in cultured murine and human cardiofibroblasts and prevented myocardial fibrosis and heart disease in both acquired and genetic models of chronic iron-overload. Iron-induced oxidative stress depletes the intrinsic antioxidant capacity and leads to formation of aggressive free radicals which impair normal cellular function. Iron-overload is associated with oxidative stress and lipid peroxidation which is a key driver of the progression of end-organ injury^16^,^18^. The potentiation of the increased expression of anti-oxidant genes in response to RSV therapy likely contributed to the enhanced anti-oxidant response in the setting of iron-overload.

In summary, our murine models of acquired and genetic iron-overload resulted in iron-overload cardiomyopathy with RSV supplementation having multiple beneficial effects. Further experimental work is needed to establish a proper dose-response relationship with RSV and the impact of concomitant iron-chelation therapy. We propose that dietary intake of RSV represents a readily available and economically feasible therapy to prevent the progression of iron-induced injury and reduce the global clinical burden of iron-overload cardiomyopathy.

## Materials and Methods

### Experimental Animal Protocols

Wild type (WT) male C57BL6 mice (from Jackson Laboratory, Bar ME) of 10–12 weeks and male HJV knockout mice (*HVJ*^*-/-*^) (kindly provided by Dr. Nancy C. Andrews, Duke University) bred in-house at the University of Alberta Health Sciences Laboratory Animal Services housing facility. WT mice were subjected to iron/placebo-injection protocol whereas HJV knockout mice (*HVJ*^*-/-*^*; HJVKO)* were treated with high iron diet (Prolab^®^RHM 3000 with iron 380 pm) respectively. All experiments were approved by Animal Care and Use Committee (ACUC) and of University of Alberta and performed in accordance to institutional guidelines, Canadian Council on Animal Care (CCAC) and the Guide for the Care and Use of Laboratory Animals published by the US National Institutes of Health (revised 2011). The iron-overload regimens used in this study corresponded to early and chronic stages of iron-overload:

1. Early stage: 5 mg of iron dextran per 25 g body weight (Sigma-Aldrich, Saint Louis, MO) or placebo (5% of dextrose with phenol) injected i.p. on a 5 day/week schedule for a total duration of 4 weeks to WT male C57BL6 mice, to study the early stage of iron-overload^16^. We also treated these early iron-overload mice with RSV enriched chow diet (Modified AIN-93G Dyets, Inc., Bethlehem, PA.) corresponding to a daily dose of 320 mg/kg for 6 weeks started at 2 weeks prior to iron injection.

2. Chronic stage: 5 mg of iron dextran per 25 g body weight injected i.p. on a 5 day/week schedule for total duration of 4 weeks followed by 1.25 mg/25 g body weight for 8 more wk in WT male C57BL6 mice. We used dietary RSV supplementation (Modified AIN-93G Diets, Inc., Bethlehem, PA) corresponding to a daily dose of 320 mg/kg^38^ in wildtype mice started at 2 weeks prior to iron injections for a total duration of 14 weeks. We also used a chronic protocol in 4 weeks old HJVKO mice by feeding them with a high iron diet (Prolab^®^RHM 3000 with iron 380 ppm) for 6 months. We also examined the effects of RSV (trans-RSV synthetic >99% pure, Lalilab Inc. Durham), on the iron-overloaded HJVKO mice by daily oral gavage (240 mg/kg/day) for 2 months starting at 4 month of age. The iron-injection protocols in WT mice was used as a model of acquired iron-overload^16^,^18^ and the HJVKO mice were used as a genetic model of hemochromatosis^24^.

### Adenoviral SERCA2 gene delivery *in vivo*

After 3 weeks of early iron-overload, mice were randomized to receive either adeno-associated virus expressing SERCA2a (AAV-9 SERCA2a, n = 10) at 1 × 10^12^ gcp/μl or adeno-associated virus expressing GFP (AAV-9 GFP, n = 5) at 5 × 10^11^ gcp/μl. The viral constructs were injected by single bolus tail vein injection method as described previously^39^,^40^ and the mice were carefully monitored for one week and their cardiac function was assessed by non-invasive echocardiography.

### Echocardiography and Invasive Hemodynamic analysis

Transthoracic echocardiography was performed on early and chronic stages of iron-overload phenotype mice with the Vevo770 high resolution imaging system equipped with a 30-MHz transducer (Visual Sonic Vevo 770) by using 1.5% isoflurane^44^,^45^. We performed PV loop analysis by using a 1.2F Scisense catheter connected to an amplifier (TCP-500 Scisense Inc.) under isoflurane anesthetic (1.5–2%) with the closed chest model as previously described^46^,^47^. Following baseline PV measurements, transient inferior vena cava occlusion was performed through the diaphragm to obtain the alteration in venous return to derive end-diastolic pressure volume relationships; transient infra-renal aortic occlusion was used to derive the end-systolic pressure volume relationship.

### Histology

Mice were anesthetized, hearts were removed and arrested in diastole by using 1M KCl, fixed with 10% buffered formalin and embedded in paraffin. Five μm thin sections were stained with Prussian blue, picro-sirius red (PSR) and trichrome stain for morphometric analysis^18^,^48^. The 5 μm tissue sections were deparaffinized in xylene and alcohol grades, then rehydrated in water and subjected to respective staining protocol as described previously^18^,^49^. The deposition of iron was visualized as blue depositions using a bright field microscope (DM 4000 B, Leica). Fibrosis pattern was evaluated by using PSR staining followed by visualization under Olympus IX81 fluorescence microscope and image analysis using MetaMorph software (Basic version 7.7.0.0.).

### Immunofluorescence

Immunofluorescence (IF) was performed on 5–10 μm thick formalin fixed and OCT embedded heart sections. Briefly, formalin fixed paraffin embedded sections were subjected to respective antigen retrieval procedures followed by blocking with blocking buffer (1% BSA in 1X PBS) for 1 hr. Similarly, OCT embedded sections were fixed with 4% paraformaldehyde for 20 min and rehydrated in 1X PBS for 30 min. Sections were then incubated with primary antibody against rat anti-mouse neutrophil (Serotec), rat anti-mouse F4/80 (Serotec), mouse anti-nitrotyrosine (Santa Cruz), mouse anti-4-HNE (Abcam), Rabbit-anti-FOXO1 (Cell Signaling), rabbit anti-collagen-I (Abcam), mouse anti-Sirt1 (Cell Signaling), mouse-anti-alpha-sarcomeric actin (Abcam), rat-anti-mouse-CD-4 (BD Pharmingen), and rat-anti-mouse-CD-8 (BD Pharmingen) over night in a humidified chamber at 4 °C. Sections were incubated with different fluorophore conjugated secondary antibodies (Invitrogen USA) as described previously^45^. The adult murine and human cardiac fibroblasts and cardiomyocytes were fixed with 4% paraformaldehyde, and then permeabilized with 0.25% Triton-X100 in PBS. Cardiac fibroblasts were then incubated with mixture of primary antibodies against alpha-smooth muscle actin (Abcam), vimentin (Abcam), FOXO1 (Cell Signaling), anti-collagen-I (Abcam) and SIRT1 (Cell Signaling) overnight at 4 °C and the cardiomyocytes were incubated separately with Nitrotyrosine (Santa Cruz) and 4-HNE (Abcam) primary antibodies at 4 °C overnight. The stained sections as well as cells were visualized under fluorescence microscopy (Olympus IX81) and quantified by using MetaMorph software.

### Dihydroethedium and Phalloidin fluorescence staining

We performed DHE fluorescent staining on 15 μm thick LV frozen sections and in cultured adult murine and human cardiomyocytes. OCT-embedded cryosections were incubated with hanks balanced salt solution (HBSS) with calcium and magnesium at 37 °C for 5 min, followed by incubation with 20 μM DHE fluorescent dye for 30 min at 37 °C. For the cultured murine and adult human cardiomyocyte were incubated for 30 min with 20 μM DHE and then washed with hanks balanced salt solution. For F-actin staining the cultured adult human cardiomyocytes were fixed in 4% paraformaldehyde, permeabilized with 0.1% Triton X-100 in Dulbecco’s phosphate buffered saline and incubated with Alexa Fluor 488 conjugated phallodin in 1% BSA for 30 minutes at room temperature. The sections were then mounted using Prolong gold antifade mounting medium with DAPI. Cardiomyocytes and LV sections were visualized using an Olympus IX81 fluorescent microscope and quantified using MetaMorph software.

### Tissue Iron Levels

20 mg frozen tissue from LV were subjected to inductive coupled plasma resonance mass spectrometry to quantify tissue iron level in the Trace Metals Laboratory, London, Western Ontario^16^,^18^. The samples were analyzed in triplicate and the average values are used.

### Measurement of Lipid Peroxidation and Glutathione levels (GSH/GSSG)

The levels of MDA, an indicator of lipid peroxidation, were measured in myocardial tissue (100–150 mg) by using a commercially available kit (Bioxytech, MDA-586TM assay, Oxis International Inc., Foster City, CA^49^,^50^. Myocardial reduced (GSH) as well as oxidized glutathione (GSSG) levels were measured as described previously^18^,^51^.

### Taqman real time PCR

mRNA expression levels were studied in iron-overload hearts and cells, by real time PCR using Taqman primers and probes (see Supplemental Table 1 for primers and probes). Total RNA was extracted from flash frozen LV-tissue by using TRIzol RNA extraction method^44^,^49^. Beside tissues RNA also extracted from cultured murine and human adult cardiomyocyte and fibroblast by using above mentioned method. 1 μg of RNA was subjected to reverse transcription to synthesize cDNA. Samples were loaded in triplicate and the data was analyzed by Light cycler® 480 system from Roche.

### Western blot analysis and Immunoprecipitation (IP)

Western analysis was performed on flash frozen LV samples as previously described^44^,^45^ using the following primary antibodies: SERCA2a (Thermo Scientific), NCX1 (Thermo scientific), collagen-I and collagen-III (Abcam), sirtuin-1 and FOXO1 (Cell signaling Inc), total and phospho (threonine-172) AMPK (Cell signaling Inc), and subsequently incubated with HRP conjugated secondary antibodies^31^. Immunoprecipitation was performed with slight modification as described previously^31^. Total protein lysate (100–200 μg) from flash frozen LV were incubated with 5 μg of anti-acetyl-Lysine (Millipore). The immune complex was captured by adding 50 μl protein A/G Plus-agarose beads (Sc-2003) with gentle rocking for 6 hr at 4 °C; the tubes were then centrifuged at 12000 g for 3 min and the supernatants were discarded. The pellets were gently washed with ice cold PBS and the immune complex resuspended in 60 μl of 2X Laemmli sample buffer and resolved on 8% SDS-PAGE then transferred to Immobilion PVDF membranes (Millipore) using a Trans-blot cell (Bio-Rad laboratories, Hercules CA, USA). Membranes were stained for total amount of protein as a loading control using MemCode (Thermo Scientific).

### Nuclear and cytosolic protein fractionation

Nuclear fractionation was performed as previously described with modifications^46^. 30 μg of nuclear protein from LV was subjected to Nrf2 (Abcam) and FOXO1 (Cell Signaling) immuno-blotting. The purity of nuclear and cytosolic fractions was verified by using Histone H3 (Cell Signaling; nuclear marker) and GAPDH (Santa Cruz; cytosolic marker).

### Isolation and culture of adult murine cardiomyocytes and cardiofibroblasts

Mice were anesthetized with inhaled isoflurane (2%) and left ventricular (LV) cardiomyocytes and cardiofibroblasts were isolated as previously described^49^,^52^. (S)-(-)-blebbistatin(1-phenyl-1,2,3,4-tetrahydro-4-hydroxypyrrolo[2,3-b]-7-methylquinolin-4-one) (25 μM) was used to inhibit myocyte contractility. Dissociated cardiomyocytes were resuspended in storage buffer (perfusion buffer pH 7.4 with 0.1% bovine serum albumin) for use in Ca^2+^ transient measurements. Dissociated myocytes were also pelleted and resuspended in perfusion buffer containing 10% FBS and exposed to sequential Ca^2+^ reintroduction. After 2 hr to allow attachment of viable myocytes, media was changed to serum free media for 24 hr with ferric ammonium citrate (FAC, Sigma, St. Louis, Mo, USA) at 145.6 μg/ml (equal to 20 μg/ml iron, 1 μg Fe = 7.28 μg FAC) and pretreated with either placebo, RSV (100 μM) or SRT1720 (1 μM) for 16 hr and during the course of exposure to FAC^53^. Cardiofibroblasts were plated onto a 10 cm culture dish in DMEM with 10% FBS, and then cultured at 37 °C. At the second passage, cardiac fibroblasts were seeded on collagen type I-treated 6-well BioFlex culture plates (Flexcell Int. Corp.), serum-deprived for 24 hr, then cyclically stretched at 10% elongation at 1 Hz and treated as described above for the cardiomyocytes.

### Isolation of and culture of adult human cardiomyocytes and cardiofibroblast

Non-failing donor human cardiomyocytes and cardiofibroblasts were isolated from human hearts obtained by the *Human Organ Procurement and Exchange* (HOPE) program. Our study was approved by the research ethics board of the University of Alberta, conformed to the principles in the Declaration of Helsinki, and written consents were obtained from all participants. LV free wall tissue (1 gm) from non-failing donor hearts was chopped into small pieces with forceps, followed by digestion in 100 mL collagenase buffer^52^,^54^,^55^ with the addition of (S)-(-)-blebbistatin (25 μM) at 37 °C. The supernatant was then centrifuged (20 G for 3 min) to pellet the cardiomyocytes and simultaneously harvest cardiofibroblasts^56^ from the supernatant, which was repeated periodically 3–4 times. Ca^2+^ reintroduction and plating were then performed as previously described^52^,^54^,^55^. The supernatant containing cardiac fibroblasts were centrifuged at 1500 rpm for 5 min and then the fibroblasts were plated in DMEM with 10% FBS, and cultured at 37 °C in a 5% CO_2_ incubator.

### Recording of Ca^2+^ transients from isolated cardiomyocytes

An aliquot of isolated cardiomyocytes was placed in a conical tube with storage solution containing 2 μM FURA2-AM and 0.04% pluronic acid and incubated at 35 °C. Cardiomyocytes were transferred to a glass-bottomed recording chamber in an inverted microscope (Olympus IX71), and allowed to settle for 5–6 min. Cells were superfused at a rate of 1.5–2 mL/min with modified Tyrode’s solution (containing in mmol/L: 135 NaCl, 5.4 KCl, 1.2 CaCl2, 1 MgCl2, 1 NaH2PO4, 10 Taurine, 10 HEPES, 10 glucose; pH 7.4 with NaOH). The perfusion solutions were heated to in-bath temperature of 35–36 °C using in-line heater (SH-27B, Harvard Apparatus) controlled by automatic temperature controller (TC-324B, Harvard Apparatus). Quiescent rod-shaped cardiomyocytes with clear striations were selected for study and fluorescence measured in response to excitation at 340 (F340) and 380 nm (F380). The F340/F380 trace was fitted from 100 to 999 ms post-electrical stimulation with a mono-exponential function to obtain the time constant (τ) and diastolic Ca^2+^ (CaD), which was defined as a value of mono-exponential fit function at 1000 ms. Systolic Ca^2+^ was defined as a maximum of the F340/F380 trace. Calculations were performed in Origin 8.5 (OriginLab) using Lab Talk custom-made scripts.

### Statistical Analysis

All data were statistically analyzed by using the SPSS Statistics 19 software and the averaged values are presented as mean ± SEM. One-way or two-way ANOVA was used for data analysis followed by multiple comparison testing using the Tukey’s test.

## Additional Information

**How to cite this article**: Das, S. K. *et al.* Iron-overload injury and cardiomyopathy in acquired and genetic models is attenuated by resveratrol therapy. *Sci. Rep.* **5**, 18132; doi: 10.1038/srep18132 (2015).

## Supplementary Material

Supplementary Information
