# Supplementary material for: Iron-overload injury and cardiomyopathy in acquired and genetic models is attenuated by resveratrol therapy
Source: Sci Rep. 2015 Dec 7;5:18132. doi: 10.1038/srep18132 (PMC4671148; doi:10.1038/srep18132)
Supplement: Supplementary Information [file srep18132-s1.pdf]

Iron-overload injury and cardiomyopathy in acquired and genetic models is attenuated by resveratrol therapy

Subhash K. Das, Wang Wang, Pavel Zhabyeyev, Ratnadeep Basu, Brent McLean, Dong Fan,
Nirmal Parajuli, Jessica DesAulniers, Vaibhav B. Patel,
Roger J. Hajjar, Jason R. B. Dyck, Zamaneh Kassiri,
and Gavin Y. Oudit

SUPPLEMENTAL MATERIAL

Supplemental Methods, Supplemental Tables 1-7 and Supplemental Figures 1-5

Supplemental Methods

Experimental Animal Protocols. Wild type (WT) male C57BL6 mice (from Jackson Laboratory, Bar ME) of 10-12 weeks and male HJV knockout mice (*$HVJ^{-/-}$*) (kindly provided by Dr. Nancy C. Andrews, Duke University) bred in-house at the University of Alberta Health Sciences Laboratory Animal Services housing facility. WT mice were subjected to iron/placebo-injection protocol whereas HJV knockout mice (*$HVJ^{-/-}$; HJVKO)* were treated with high iron diet (Prolab®RHM 3000 with iron 380 ppm) respectively. The iron-overload regimens used in this study corresponded to early, chronic and advanced stages of iron-overload:

1. **Early stage:** 5 mg/25 gm body weight of iron dextran (from sigma) or placebo (5% of dextrose with phenol) i.p injected on a 5day/week schedule for a total duration of 4 weeks to WT male C57BL6 mice, to study the early stage of iron-overload.[1] We also treated these early iron-overload mice with resveratrol enriched chow diet (Modified AIN-93G Dyets, Inc., Bethlehem, PA., 320 mg/kg/day) for 6 weeks started at 2 weeks prior to iron injection.
2. **Chronic stage:** 5 mg/25 gm body weight of iron-dextran i.p. was injected on a 5 day/week schedule for total duration of 4 weeks followed by 1.25 mg/25 gm body weight for 8 more weeks in WT male C57BL6 mice. We used dietary resveratrol supplementation (Modified AIN-93G Diets, Inc., Bethlehem, PA.320 mg/kg/day corresponding to plasma levels of 10-20 µmol/L in rodents)[2] in wild type mice for 14 weeks. We also used a chronic protocol in 4 weeks old HJVKO mice by feeding them with a high iron diet (Prolab®RHM 3000 with iron 380 ppm) for 6 months. We also examined the effects of resveratrol (trans-resveratrol synthetic >99% pure, Lalilab Inc. Durham), on the iron-overloaded HJVKO mice by daily oral gavage (240 mg/kg/day) for two months starting at 4 mths of age. The iron-injection protocols in WT mice was used as a model of acquired iron-overload[1] and the HJVKO mice were used as a genetic model of hemochromatosis.[3]
3. **Advanced stage:** We also treated 4 week old HJVKO mice with high iron diet until they were 1 year old. We also examined the effects of resveratrol on the 1 year old iron-overloaded HJVKO mice by daily oral gavage (240

mg/kg/day) for three months starting at 9 months of age. We dissolved resveratrol in 5.4% ethanol/corn oil.[4-6] The placebo control groups received 5.4% ethanol/corn oil.

Adenoviral SERCA2 gene delivery *in vivo*. After 3 weeks of early iron-overload, mice were randomized to receive either adeno-associated virus expressing SERCA2a (AAV-9 SERCA2a, n=10) at $1x10^{12}$ gcp/µl or adeno-associated virus expressing GFP (AAV-9 GFP, n=5) at $5x10^{11}$ gcp/µl. The viral constructs were injected by single bolus tail vein injection method as described previously[7, 8] and the mice were carefully monitored for one week and their cardiac function was assessed by the non-invasive echocardiography.

Echocardiography. Transthoracic echocardiography was performed on early, chronic and advanced stages of iron-overload phenotype mice with the Vevo770 high resolution imaging system equipped with a 30-MHz transducer (Visual Sonic Vevo 770) by using 0.8% isoflurane.[9, 10] Both diastolic as well as systolic, cardiac function parameters are recorded and analyzed. Systolic function was assessed using B-mode and M-mode images of Echocardiography. M-mode images were obtained for measurements of left ventricular (LV) wall thickness, LV end-diastolic diameter (LVEDD), and LV end-systolic diameter (LVESD) (measures of LV dilation). LV fractional shortening (FS) and LV ejection fraction (EF) were calculated using the following equations: FS (%) = (LVEDD-LVESD/LVEDD) ×100 and EF (%) = (LVEDV-LVESV/LVEDV) ×100. Diastolic function was assessed using pulsed-wave Doppler imaging of the trans-mitral filling pattern with the early trans-mitral filling wave (E-wave) followed by the late filling wave due to atrial contraction (A-wave). Isovolumetric relaxation time (IVRT) was calculated as the time from closure of the aortic valve to initiation of the E-wave. The deceleration time of the E-wave (DT) was determined by measuring the time needed for the down-slope of the peak of the E-wave to reach the baseline, while the rate of E-wave deceleration rate (EWDR) was calculated as the E-wave divided by the DT. Tissue Doppler imaging (TDI) represents a novel and validated technique to assess systolic and diastolic function, with a reduction in $E^{'}$ and an elevation in $E/E^{'}$ being considered as valid markers of elevated LV filling pressure and diastolic dysfunction. TDI was carried out at the inferolateral region in the radial short axis at the base of the LV with the assessment of peak annular systolic ($S^{'}$), early diastolic ($E^{'}$), and late diastolic ($A^{'}$) myocardial velocities.

Invasive hemodynamic analysis. We performed PV loop analysis by using 1.2F Scisense catheter connected to an amplifier (TCP-500 Scisense Inc.).[11] Mice were anesthetized by using isoflurane (1-1.5%) and are maintained at 37^{0}C by using heating pad. An incision was made in the right common carotid artery and the catheter was carefully inserted into the incision and the catheter was advanced through the aortic valve and placed into the LV chamber. The position of the catheter was monitored by pressure along with the magnitude and phase using ADvantage pressure volume system (Scisense Inc., London, Canada) and iworx (iWorx Systems Inc., Dover, USA) data acquisition system connected to the catheter. Initially, the catheter position was set in the LV to obtain the magnitude difference of more than 200µS along with a physiological pressure-volume loop shape. After the magnitude was accomplished in the desired range, the phase was adjusted to 4-8 with slightly adjusting the position of the catheter in the LV where phase represents the conductivity imparted by the LV tissue. Once, the desired range for magnitude and phase was achieved, baseline scan was performed to derive volume using Baan's equation and pressure-volume loop was obtained using the LabScribe2 software (version 2.347000). Following baseline PV measurements, transient inferior vena cava occlusion was performed through the diaphragm to obtain the alteration in venous return to derive end-diastolic pressure volume relationships; transient infra-renal aorta occlusion was used to derive the end-systolic pressure volume relationship. Load-dependent and load-independent indices of LV functions were derived. By making and plotting the instantaneous values of pressure and volume at different time points, we determined the ESP, EDP, ESV and EDV in mmHg and µL respectively. Heart rate was estimated beat-to-beat cycle length. The SV =EDV-ESV, CO=SVxHR, EF%=SV/EDV, the SW is the area under a PV-Loop and was normalized by dividing with EDV to obtain preload recruitable stroke work (PRSW) which is also a load independent parameter. $+dP/dt_{(max),}$ and $-dP/dt_{(min)}$ are the first derivative of pressure with respective to time and we also took their ratio ($-dP/dt_{(min)}$ /$+dP/dt_{(max)}$) to show a better index of relaxation phase, tau(τ)-the time constant of monoexponential pressure decay during isovolumic relaxation, the preload-independent $+dP/dt_{(max),}$ also called the starlings contractile index (SCI) was calculated by dividing $+dP/dt_{(max)}$/EDV, which is a better index of myocardial contractility. Systolic and diastolic cardiac performances were also assessed by the end-systolic pressure volume relationship (ESPVR) and end-diastolic pressure volume relationship (EDPVR), respectively.

Histology. Mice were anesthetized and the hearts (arrested in diastole by using 1M KCl) were removed and fixed with 10% buffered formalin and embedded in paraffin. Five µm thin sections were stained with Prussian blue, picro-sirius red (PSR) and trichrome stain for morphometric analysis.[1, 12] The 5 µm tissue sections were deparaffinized in xylene and alcohol grades, then rehydrated in water and subjected to respective staining protocol as described previously.[12, 13] The deposition of iron was visualized as blue depositions using bright field microscope. Fibrosis pattern was evaluated by using PSR staining followed by visualization under Olympus IX81 microscope and image analysis using MetaMorph software.

Immunofluorescence. Immunofluorescence (IF) was performed on 5-10µm thick formalin fixed and OCT embedded sections from hearts. Briefly, formalin fixed paraffin embedded sections were deparaffinized in xylene and alcohol grades, then rehydrated in water and subjected to respective antigen retrieval procedures followed by blocking with blocking buffer (1% BSA in 1X PBS) for 1 hour. Similarly the OCT embedded sections were fixed with 4% paraformaldehyde for 20 min and rehydrated in 1X PBS for 30 min. The paraformaldehyde fixed OCT sections were then permeabilized with 0.25% Triton-X-100 for 15 min followed by blocking with blocking buffer (1% BSA in 1X PBS) for 1 hr and then incubated with the corresponding primary antibody: rat anti-mouse neutrophil (Serotec:MCA771GA), rat anti-mouse F4/80 (Serotec:MCA497GA), mouse anti-nitrotyrosine (Santa Cruz), mouse anti-4-HNE (Abcam), Rabbit-anti-FOXO1 (Cell Signaling-2880), rabbit anti-collagen-I (Abcam-ab84956), Mouse anti-Sirt1 (Cell Signaling-8469), mouse-anti-alpha-sarcomeric actin (Abcam#ab-28052), rat-anti-mouse-CD-4 (BDPharmingen™:550954), and rat-anti-mouse-CD-8 (BDPharmingen™ : 550281) in a humidified chamber at 4^0C. Sections were incubated with different fluorophore conjugated secondary antibodies (Invitrogen USA) respectively as described previously.[10, 14-16] The adult murine and human cardiac fibroblasts and cardiomyocytes after their respective treatment were washed several times with PBS and fixed with 4% paraformaldehyde for 20 mins, both cardiomyocytes and cardiac fibroblasts were then permeabilized with 0.25% Triton-X100 in PBS for 5 min, followed by incubation with 1% bovine serum albumin. The cardiac fibroblasts were then incubated with mixture of primary antibodies against alpha-smooth muscle actin(Abcam-5694) (1:100), vimentin (1:1000), FOXO1(Cell Signaling-2880) (1:100), anti-collagen-I (Abcam-ab84956), (1:300) and SIRT1(Cell Signaling-8469) (1:100) overnight at 4^0C and the cardiomyocytes were incubated separately with Nitrotyrosine (Santa Cruz), (1:400), 4-HNE (Abcam) (1:600) primary antibodies at 4^0C overnight. Both cultured cell types were incubated with mixture of different fluorophore conjugated secondary antibodies for 1 hr at 37^0C (Invitrogen). Above mentioned tissue sections as well cells were washed with 1X PBS, three times for 5 min each, in between each steps, after mounting with Prolong Gold antifade mounting medium with DAPI and some sections without DAPI (Invitrogen). The stained sections as well as cells were visualized under fluorescence microscope (Olympus IX81) and quantified by using MetaMorph software.

Dihydroethedium and Phalloidin fluorescence staining. Dihydroethedium (DHE) is an ROS detection fluorescent dye which produces excess fluorescent signal in presence of ROS. Phalloidin stabilizes the F-actin and fluorophore conjugated phalloidin can be used to stains the cytoskeletal F-actin. We performed dihydroethedium fluorescent staining on 15µm thick LV frozen sections and also in cultured adult murine and human single cell cardiomyocytes. OCT-embedded cryosections were incubated with the 1X hanks balanced salt solution (HBSS) with calcium and magnesium at 37^0C for 5 min, followed by incubation with 20µM DHE fluorescent dye in 1X HBSS for 30 min at 37^0C. For the cultured murine and adult human cardiomyocyte were incubated for 30 min with 20µM DHE and then washed with 1X HBSS. For F-actin staining the cultured adult human cardiomyocytes were fixed in 4% paraformaldehyde, permeabilized with 0.1% Triton X-100 in Dulbecco's phosphate buffered saline (DPBS) and incubated with Alexa Fluor 488 conjugated phallodin in 1% BSA for 30 minutes at room temperature. The sections were then mounted using Prolong gold antifade mounting medium with DAPI. The sections were washed three times with DPBS in between each step. The tissue sections and cultured cardiomyocyte dishes were wrapped with foil to minimize light exposure and visualized under Olympus IX81 fluorescent microscope and quantified by using MetaMorph software.

Tissue iron levels. 20 mg frozen tissue samples from the LV were subjected to inductive coupled plasma resonance mass spectrometry to quantify tissue iron level in the Trace Metals Laboratory, London, Western Ontario.[1, 12] The samples were analyzed in triplicate and the average values are used.

Measurement of lipid peroxidation. The levels of malondialdehyde (MDA), an indicator of lipid peroxidation, were measured in myocardial and hepatic tissues (100-150mg) by using a commercially available kit

(Bioxytech@ MDA-586TM assay kit cat#21044, OxiResearch, Percipio Biosciences Inc. Los Angeles, CA U.S.A).[13, 17] Briefly, tissue samples were homogenized in potassium phosphate extraction (KPE) buffer pH 7.5 (0.1M potassium phosphate, 5mM EDTA, 0.1% Triton X-100 and 0.6% sulfosalicylic acids) containing 5mM BHT. The samples were allowed to react with N-methyl-2-phenylindole (NMPI) in the acidic pH at 45°C for 1 hour. The clear supernatant were then collected and read in at 586 nm using a plate reader (Spectramax M5, Molecular Devices, and Sunnyvale, CA). MDA levels were estimated using a standard curve derived using 0.5 to 4.0 µM of standard MDA.

Taqman real time PCR. mRNA expression levels were studied in iron-overload hearts, by real time PCR using Taqman primers and probes (see Table of primers and probes listed below). Total RNA was extracted from flash frozen LV-tissue and hepatic tissue by using TRIzol RNA extraction method[9]. Beside tissues RNA also extracted from cultured murine and human adult cardiomyocyte and fibroblast by using above mentioned method. 1µg of RNA was subjected to reverse transcription to synthesize cDNA. Real time PCR was performed with 5µl of suitable cDNA dilutions from unknown and standard (brain cDNA), 8µl Taqman master-mix (includes-primers + Probes) were loaded on white 384 Light cycler®480 multi well plates supplied from Roche with 18s rRNA as internal control. Samples were loaded in triplicate and the data was analyzed by Light cycler® 480 system from Roche.

Measurement of Glutathione levels (GSH/GSSG). Myocardial and hepatic reduced (GSH) as well as oxidized glutathione (GSSG) levels were measured as described previously.[12, 18]

Western blot. Protein lysates (30-100µg) from flash frozen LV tissue were resolved on 8%, 15% sodium dodecyl sulphate-poly acrylamide gel electrophoresis (SDS-PAGE) then transferred to PVDF membranes using a Trans-blot cell (Bio-Rad laboratories, Hercules CA USA) as previously described.[9, 10] The membranes were blocked with 5% milk, incubated with respective primary antibodies including SERCA2a, NCX1, collagen-I and collagen-III, sirtuin-1, FOXO1, total and phospho (threonine-172) AMPK, and subsequently incubated with HRP conjugated secondary antibodies respectively.[19, 20] Blots were scanned and quantified by using ImageQuant LAS 4000 (GE Health care, Biosciences).

Immunoprecipitation (IP). Immunoprecipitation was performed with slight modification as described previously.[19, 21] Total protein lysate (100-200 µg) from flash frozen LV tissue were incubated with 5 µg of anti-acetyl-Lysine (Millipore #05-515) overnight at 4^0C with gentle rock. The immune complex was captured by adding 50µl protein A/G Plus-agarose beads (Sc-2003) with gentle rocking for 6 hours at 4^0C. After that tubes were centrifuged at 12000g for 3 minutes and the supernatants were discarded. The pellets were gently washed with ice cold 1X PBS and resuspended the immune complex in 60µl of 2X Laemmli sample buffer and resolved on 8% sodium dodecyl sulphate-poly acrylamide gel electrophoresis (SDS-PAGE) then transferred to PVDF membranes using a Trans-blot cell (Bio-Rad laboratories, Hercules CA USA).

Nuclear and cytosolic protein fractionation. Nuclear fractionation was performed as previously described with modifications[11]. Briefly, LV tissues were homogenized in hypotonic lysis buffer (10 mM K-HEPES (pH 7.9), 1.5 mM MgCl2, 10mM KCl, 1 mM DTT, 0.2 mM Na_3VO_4, 1X protease inhibitor cocktail (Calbiochem), 1X phosphatase inhibitors (Sigma and Calbiochem). The total homogenate was centrifuged at 100 g for 5 minutes to collect unbroken tissues. The supernatant was then centrifuged at 2,000 g for 10 minutes to precipitate crude nuclei from cell membrane and cytosolic proteins (second supernatant). The second supernatant was further centrifuged at 100,000 g for 90 minutes to separate soluble cytosolic proteins (third supernatant) from membrane pellet. The crude nuclear fraction was resuspended in hypotonic lysis buffer supplemented with 2.4M sucrose, and then layered on top of a 2.4M sucrose cushion and purified by centrifugation at 100,000 g for 90 minutes. Following ultracentrifugation, the purified nuclear pellet was resuspended in storage buffer (20 mM Na-HEPES (pH 7.9), 0.42 M NaCl, 1.5 mM MgCl2, 0.2 mM EDTA, 0.2 mM EGTA, 0.5 mM PMSF, 0.5 mM DTT, 25% Glycerol, 1 x protease and phosphatase inhibitors). 30µg of nuclear protein from LV was subjected to Nrf2 (Abcam ab53019) and FOXO1 (Cell Signaling-2880) immuno-blotting. The purity of nuclear and cytosolic fractions was verified by using Histone H3 (Cell Signaling; nuclear marker) and GAPDH (Santa Cruz; cytosolic marker).

Isolation and culture of adult murine cardiomyocytes. Adult murine left ventricular (LV) cardiomyocytes were isolated as previously described.[22] (S)-(-)-Blebbistatin(1-phenyl-1,2,3,4-tetrahydro-4-hydroxypyrrolo[2,3-b]-7-

methylquinolin-4-one) (25µM) from Toronto Research Chemicals was used to inhibit myocyte contractility by selective inhibition of ATPase activity of myosin-II as previously reported.[23] Briefly, mice were injected with 0.2 mL of 100 IU/mL heparin for 15 min and then anesthetised using 2% isoflurane (1 L/min oxygen flow rate) provided through a nose cone. After opening the chest cavity, the heart was quickly excised, cannulated, and attached to the perfusion system. Following 3 min of perfusion with perfusion buffer (containing in mmol/L: 120 NaCl, 14.7 KCl, 1.2 KH2PO4, 10 HEPES, 1.2 MgSO4, 10 NaHCO3, 10 taurine, 20 glucose; pH 7.0), the heart was digested with 2.4 mg/mL collagenase type 2 (Worthington) for 7-8 min. After sufficient digestion, the ventricles were removed, dissociated using forceps and transfer pipettes, and resuspended in storage buffer (perfusion buffer pH 7.4 with 0.1% bovine serum albumin) for use in Ca^{2+} transient measurements. Alternately, for culture of myocytes, all steps after perfusion were performed in a laminar flow culture hood. Dissociated myocytes were pelleted (20g for 3min) and resuspended in perfusion buffer containing 10% FBS and 12.5µM Ca^{2+}. Sequential Ca^{2+} reintroduction was performed at 5min intervals to raise Ca^{2+} levels to 100µM, 400µM and finally 900µM. Myocytes were then pelleted (20g for 3min) and resuspended in Minimal Essential Media (Sigma, M4780) containing 10% FBS and Penicillin-Streptomycin (Gibco, 15140) and plated on laminin coated dishes. After 2hrs to allow attachment of viable myocytes, media was changed to serum free media for 12 hours with ferric ammonium citrate (FAC, Sigma, St. Louis, Mo, USA) at 145.6µg/ml (equal to 20µg/ml iron, 1µg Fe=7.28µg FAC) in the absence or presence of resveratrol (22.8 mg/ml).[24, 25]

Isolation and culture of adult murine cardiofibroblasts. The standard method of cardiac fibroblast isolation and culture from adult murine left ventricle was done as previously described.[22] Briefly, 10 wk old mice were injected with 0.05 mL of 1000USP/mL heparin for 15 min and then anesthetized. Then the heart was quickly excised and perfused at a constant speed (4ml/min) within 45s, and digested with collagenase type 2 (Worthington). After digestion, the ventricles were removed, dissociated using forceps and transfer pipettes in stopping buffer (10% FBS perfusion buffer). Cardiomyocytes were collected by centrifugation at 20g for 3 min. The supernatant containing cardiac fibroblasts were centrifuged at 1500 rpm for 5 min to harvest the fibroblasts, and then the fibroblasts were plated onto a 10 cm culture dish in DMEM with 10% FBS, and then cultured at 37°C in a 5% CO2 incubator. At the second passage, cardiac fibroblasts were seeded onto collagen type I-treated 6-well BioFlex culture plates (Flexcell Int. Corp.). Cardiac fibroblasts were serum-deprived for 24 hr and then cyclically stretched at 10% elongation at 1Hz. Fibroblasts were exposed to ferric ammonium citrate (FAC, Sigma, St. Louis, Mo, USA) at 145.6 µg/ml (equal to 20µg/ml iron, 1µg Fe=7.28µg FAC) for 24 hr and pretreated with either placebo or resveratrol (100 µM) for 16 hours and during the course of exposure to FAC.

Isolation of adult human cardiomyocytes and cardiofibroblast. Non-failing donor human cardiomyocytes and cardiofibroblasts were similarly isolated, with the following modification: approximately 1 gram of tissue from the LV free wall of healthy donor hearts was chopped into small pieces with forceps, followed by digestion in 100mL collagenase buffer[22, 26, 27] with the addition of (S)-(-)-Blebbistatin (25µM) at 37°C and mixed with a magnetic stir bar. After 20 min, stirring was periodically stopped to allow large chunks to settle. The supernatant was then centrifuged (20g for 3 min) to pellet the cardiomyocytes and simultaneously harvest the cardiac fibroblast[28] from the supernatant, and the remaining digestion buffer was returned to the digestion beaker and stirring continued. This was repeated periodically 3-4 times, ended after a total digestion time of approximately 60 minutes. Ca^{2+} reintroduction and plating were then performed as previously described.[22, 26, 27] The supernatant containing cardiac fibroblasts were centrifuged at 1500 rpm for 5 min to harvest the fibroblasts, and then the fibroblasts were plated onto a 10 cm culture dish in DMEM with 10% FBS, and then cultured at 37°C in a 5% CO2 incubator. Following a similar protocol for the murine cardiomyocytes and cardiofibroblasts, cells were treated with ferric ammonium citrate (FAC, Sigma, St. Louis, Mo, USA) at 145.6µg/ml (equal to 20µg/ml iron, 1µg Fe=7.28µg FAC) in the absence or presence of resveratrol (22.8 mg/ml).[24, 25]

Recording of Ca^{2+} transients from isolated cardiomyocytes. An aliquot of isolated cardiomyocytes was placed in a conical tube with storage solution containing 2µmol/l FURA2-AM and 0.04% pluronic acid and incubated at 35 °C. After 15 min, solution was carefully aspirated and replaced with storage solution, and cells were incubated at 35 °C for another 15 min. Following this, cell were collected from the bottom of the tube, transferred to a glass-bottomed recording chamber on top of inverted microscope (Olympus IX71), and allowed to settle for 5-6 min. Cells were superfused at a rate of 1.5-2 mL/min with modified Tyrode's solution (containing in mmol/L: 135 NaCl, 5.4 KCl, 1.2 CaCl2, 1 MgCl2, 1 NaH2PO4, 10 Taurine, 10 HEPES, 10 glucose; pH 7.4 with NaOH). The

perfusion solutions were heated to in-bath temperature of 35-36°C using in-line heater (SH-27B, Harvard Apparatus) controlled by automatic temperature controller (TC-324B, Harvard Apparatus). Quiescent rod-shaped cardiomyocytes with clear striations were selected for study. Platinum-wire electrodes were placed near the cell just outside of the microscope view at 400X magnification. Cardiomyocytes were paced with 3-4 V (50% above threshold) and pulse duration of 2.5ms using S48 stimulator (Grass Technology). Fluorescence signal was recorded at 510-nm emission frequency using ratiometric fluorometer D-104 (Photon Technology International) in response to excitation frequencies of 340 and 380 nm injected via 40X objective (UAPO 40X3/340, Olympus). Excitation frequencies were alternated at a frequency of 200 Hz. Fluorescence in response to excitations were recorded for 60s and stored on hard drive for offline analysis. The 60-s recording (sweep) consisted of 5-s baseline (no electrical stimulation) and 55-s stimulation at 1 Hz (i.e., 55 pulses in total). 5 to 10 sweeps with in-between rest (no stimulation) of 2 min were recorded per cardiomyocyte. At the end of the sweep series for a cardiomyocyte, background signal was recorded for adjustment during analysis. Signal from 1-2 myocytes were recorder from one loading cycle. Sweeps showing stable regular response to electrical stimulation were selected for analysis. Analysis consisted of signal processing to reduce noise in the signal and measurement of parameters of interest (diastolic Ca^{2+} or CaD, systolic Ca^{2+} or CaS, amplitude of the transient or ΔCa (CaS-CaD), and time constant of the transient or τ). Signal processing consisted of (i) background adjustment and (ii) averaging of fluorescence signal for stimulation pulses from 20th to 55th within the train. These operation were performed separately for F340 (fluorescence in response to excitation at 340 nm) and F380 (fluorescence in response to excitation at 380 nm). After that, signal was expressed as F340/F380 to estimate intracellular Ca^{2+} concentration. F340/F380 trace was fitted from 100 to 999 ms post-electrical stimulation with a mono-exponential function to obtain time constant (τ) and diastolic Ca^{2+} (CaD), which was defined as a value of mono-exponential fit function at 1000 ms (since the fitted trace is an average of steady-state response to electrical stimulation, end-value of a transient is equivalent to the pre-stimulation value of the next transient). Systolic Ca^{2+} was defined as a maximum of F340/F380 trace. To minimize contribution of the residual noise, 5-point adjacent average was calculated prior finding the maximum. Calculations were performed in Origin 8.5 (OriginLab) using Lab Talk custom-made scripts.

Statistical Analysis: All data were statistically analyzed by using the SPSS Statistics 19 software and the averaged values are presented as mean ± SEM. One-way or two-way ANOVA was used for data analysis followed by multiple comparison testing using the Tukey’s test.

References

1. Oudit GY, Sun H, Trivieri MG, Koch SE, Dawood F, Ackerley C, *et al.* L-type Ca2+ channels provide a major pathway for iron entry into cardiomyocytes in iron-overload cardiomyopathy. *Nat Med* 2003;**9**:1187-1194.
2. Dolinsky VW, Jones KE, Sidhu RS, Haykowsky M, Czubryt MP, Gordon T, *et al.* Improvements in skeletal muscle strength and cardiac function induced by resveratrol during exercise training contribute to enhanced exercise performance in rats. *J Physiol* 2012;**590**:2783-2799.
3. Huang FW, Pinkus JL, Pinkus GS, Fleming MD, Andrews NC. A mouse model of juvenile hemochromatosis. *J Clin Invest* 2005;**115**:2187-2191.
4. Sale S, Verschoyle RD, Boocock D, Jones DJ, Wilsher N, Ruparelia KC, *et al.* Pharmacokinetics in mice and growth-inhibitory properties of the putative cancer chemopreventive agent resveratrol and the synthetic analogue trans 3,4,5,4'-tetramethoxystilbene. *Br J Cancer* 2004;**90**:736-744.
5. Amri A, Chaumeil JC, Sfar S, Charrueau C. Administration of resveratrol: What formulation solutions to bioavailability limitations? *Journal of controlled release : official journal of the Controlled Release Society* 2012;**158**:182-193.
6. Johnson JJ, Nihal M, Siddiqui IA, Scarlett CO, Bailey HH, Mukhtar H, *et al.* Enhancing the bioavailability of resveratrol by combining it with piperine. *Molecular nutrition & food research* 2011;**55**:1169-1176.
7. Cutler MJ, Wan X, Plummer BN, Liu H, Deschenes I, Laurita KR, *et al.* Targeted sarcoplasmic reticulum Ca2+ ATPase 2a gene delivery to restore electrical stability in the failing heart. *Circulation* 2012;**126**:2095-2104.
8. Hajjar RJ. Potential of gene therapy as a treatment for heart failure. *J Clin Invest* 2013;**123**:53-61.
9. Zhong J, Basu R, Guo D, Chow FL, Byrns S, Schuster M, *et al.* Angiotensin-converting enzyme 2 suppresses pathological hypertrophy, myocardial fibrosis, and cardiac dysfunction. *Circulation* 2010;**122**:717-728.
10. Patel VB, Bodiga S, Basu R, Das SK, Wang W, Wang Z, *et al.* Loss of angiotensin-converting enzyme-2 exacerbates diabetic cardiovascular complications and leads to systolic and vascular dysfunction: a critical role of the angiotensin II/AT1 receptor axis. *Circ Res* 2012;**110**:1322-1335.
11. Wang W, McKinnie SM, Patel VB, Haddad G, Wang Z, Zhabyeyev P, *et al.* Loss of Apelin Exacerbates Myocardial Infarction Adverse Remodeling and Ischemia-reperfusion Injury: Therapeutic Potential of Synthetic Apelin Analogues. *Journal of the American Heart Association* 2013;**2**:e000249.
12. Oudit GY, Trivieri MG, Khaper N, Husain T, Wilson GJ, Liu P, *et al.* Taurine supplementation reduces oxidative stress and improves cardiovascular function in an iron-overload murine model. *Circulation* 2004;**109**:1877-1885.
13. Patel VB, Wang Z, Fan D, Zhabyeyev P, Basu R, Das SK, *et al.* Loss of p47phox subunit enhances susceptibility to biomechanical stress and heart failure because of dysregulation of cortactin and actin filaments. *Circ Res* 2013;**112**:1542-1556.
14. Basu R, Fan D, Kandalam V, Lee J, Das SK, Wang X, *et al.* Loss of Timp3 gene leads to abdominal aortic aneurysm formation in response to angiotensin II. *J Biol Chem* 2012;**287**:44083-44096.
15. Supale S, Thorel F, Merkwirth C, Gjinovci A, Herrera PL, Scorrano L, *et al.* Loss of prohibitin induces mitochondrial damages altering beta-cell function and survival and is responsible for gradual diabetes development. *Diabetes* 2013;**62**:3488-3499.

16. Patel VB, Bodiga S, Fan D, Das SK, Wang Z, Wang W, *et al.* Cardioprotective effects mediated by angiotensin II type 1 receptor blockade and enhancing angiotensin 1-7 in experimental heart failure in angiotensin-converting enzyme 2-null mice. *Hypertension* 2012;**59**:1195-1203.
17. Erdelmeier I, Gerard-Monnier D, Yadan JC, Chaudiere J. Reactions of N-methyl-2-phenylindole with malondialdehyde and 4-hydroxyalkenals. Mechanistic aspects of the colorimetric assay of lipid peroxidation. *Chemical research in toxicology* 1998;**11**:1184-1194.
18. Rahman I, Kode A, Biswas SK. Assay for quantitative determination of glutathione and glutathione disulfide levels using enzymatic recycling method. *Nat Protoc* 2006;**1**:3159-3165.
19. Brunet A, Sweeney LB, Sturgill JF, Chua KF, Greer PL, Lin Y, *et al.* Stress-dependent regulation of FOXO transcription factors by the SIRT1 deacetylase. *Science* 2004;**303**:2011-2015.
20. Ennis IL, Li RA, Murphy AM, Marban E, Nuss HB. Dual gene therapy with SERCA1 and Kir2.1 abbreviates excitation without suppressing contractility. *The Journal of clinical investigation* 2002;**109**:393-400.
21. Ching LC, Kou YR, Shyue SK, Su KH, Wei J, Cheng LC, *et al.* Molecular mechanisms of activation of endothelial nitric oxide synthase mediated by transient receptor potential vanilloid type 1. *Cardiovascular research* 2011;**91**:492-501.
22. O'Connell TD, Rodrigo MC, Simpson PC. Isolation and culture of adult mouse cardiac myocytes. *Methods Mol Biol* 2007;**357**:271-296.
23. Kabaeva Z, Zhao M, Michele DE. Blebbistatin extends culture life of adult mouse cardiac myocytes and allows efficient and stable transgene expression. *American journal of physiology Heart and circulatory physiology* 2008;**294**:H1667-1674.
24. Parkes JG, Liu Y, Sirna JB, Templeton DM. Changes in gene expression with iron loading and chelation in cardiac myocytes and non-myocytic fibroblasts. *J Mol Cell Cardiol* 2000;**32**:233-246.
25. Liu Y, Templeton DM. Iron-loaded cardiac myocytes stimulate cardiac myofibroblast DNA synthesis. *Molecular and cellular biochemistry* 2006;**281**:77-85.
26. del Monte F, Harding SE, Schmidt U, Matsui T, Kang ZB, Dec GW, *et al.* Restoration of contractile function in isolated cardiomyocytes from failing human hearts by gene transfer of SERCA2a. *Circulation* 1999;**100**:2308-2311.
27. Bird SD, Doevendans PA, van Rooijen MA, Brutel de la Riviere A, Hassink RJ, Passier R, *et al.* The human adult cardiomyocyte phenotype. *Cardiovasc Res* 2003;**58**:423-434.
28. Kawano H, Do YS, Kawano Y, Starnes V, Barr M, Law RE, *et al.* Angiotensin II has multiple profibrotic effects in human cardiac fibroblasts. *Circulation* 2000;**101**:1130-1137.

Supplemental Table 1. List of Taqman Primers and Probes.

**Gene**	**Type**	**Sequence**
ANF	Forward:<br>Reverse:<br>Probe:	5'-GGA GGA GAA GAT GCC GGT AGA-3'<br>5'-GCT TCC TCA GTC TGC TCA CTC A-3'<br>5'-FAM-TGA GGT CAT GCC CCC GCA GG-TAMRA-3’
BNP	Forward:<br>Reverse:<br>Probe:	5'-CTG CTG GAG CTG ATA AGA GA-3'<br>5'-TGC CCA AAG CAG CTT GAG AT-3'<br>5'-FAM-CTC AAG GCA GCA CCC TCC GGG-TAMRA-3'
β- MHC	Forward:<br>Reverse:<br>Probe:	5'-GTGCCA AGG GCC TGA ATG AG-3'<br>5'-GCA AAG GCT CCA GGT CTG A-3'<br>5'-FAM-ATC TTG TGC TAC CCA GCT CTA A-TAMRA-3'
IL-6	Forward:<br>Reverse:<br>Probe:	5'-ACAACCACGGCCTTCCCTACTT-3’<br>5'-CACGATTTCCCAGAGAACATGTG-3’<br>5'-FAM-TTCACAGAGGATACCACTCCCAACAGACCT-TAMRA-3’
IL-1β	Forward:<br>Reverse:<br>Probe:	5'-AACCTGCTGGTGTGTGACGTTC-3’<br>5'-CAGCACGAGGCTTTTTTGTTGT-3’<br>5'- FAM-TTAGACAGCTGCACTACAGGCTCCGAGATG-TAMRA-3’
TNF-α	Forward:<br>Reverse:<br>Probe:	5'- ACAAGGCTGCCCCGACTAC-3’<br>5'- TTTCTCCTGGTATGAGATAGCAAATC-3’<br>5'-FAM-TGCTCCTCACCCACACCGTCAGC-TAMRA-3’
Pro-Collagen-I	Forward:<br>Reverse:<br>Probe:	5’- CTTCACCTACAGCACCCTTGTG-3’<br>5’-TGACTGTCTTGCCCCAAGTTC-3’<br>5’-FAM-CTGCACGAGTCACACC-TAMRA-3'
Pro-Collagen-III	Forward:<br>Reverse:<br>Probe	5’- TGTCCTTTGCGATGACATAATCTG -3’<br>5’- AATGGGATCTCTGGGTTGGG-3’<br>5’-FAM- ATGAGGAGCCACTAGACT-TAMRA-3' □
TGF-β	Forward:<br>Reverse:<br>Probe	5’- CCTGCAAGACCATCGACATG-3’<br>5’- ACAGGATCTGGCCACGGAT-3’<br>5’-FAM-CTGGTGAAACGGAAGCGCATCGAA-TAMRA-3'
SOD1	Premix	Mm01700393_g1
CAT	Premix	Mn00437992_m1

Hmox-1	Premix	Mn00516005_m1
Trfc(Transferrin R)	premix	Mm00441941_m1
HJV(Hemojuvelin)	Premix	Mm00510148_s1
FPN1(Ferroportin)	Premix	Mm00489837_m1
HAMP1(Hepcidin1)	Premix	Mm00519025_m1
HAMP2(Hepcidin2)	Premix	Mm00842044_g1
Ftl1(Ferritin-L)	Premix	Mm03030144_g1
Fth1(Ferritin-H)	Premix	Mm00085707_g1
ATP2A2(SERCA2A)	Premix	Mm01201431_m1
ATP2A2(hSERCA2A)	Premix	Hs00544877_m1
Col1A1(Human Pro-Collagen-I)	Premix	Hs00164004_m1
Col3A1(Human Pro-Collagen-III)	Premix	Hs00943809_m1
ACTA2 (Human α-SMA)	Premix	Hs00426835_g1
Human TGF-β	Forward:<br>Reverse:<br>Probe:	5’-GTGACAGCAGGGATAACACACTG-3’<br>5’-CATGAATGGTGGCCAGGTC-3’<br>5’-FAM-ACATCAACGGGTTCACTACCGGC-TAMRA-3’
HPRT	Forward:<br>Reverse:<br>Probe	5'- AGCTTGCTGGTGAAAAGGAC-3’<br>5'- CAACTTGCGCTCATCTTAGG-3’<br>5’-FAM-CAACAAAGTCTGGCCTGTATCCAAC-TAMRA-3’
18S rRNA	Premix	Mm03928990_g1
Human 18S rRNA		Catalogue#4308329 (Invitrogen TM)

Supplemental Table 2. Echocardiographic assessment of cardiac function in early iron-overloaded male WT mice.

	WT+Placebo	WT+Iron	WT+Iron+Resveratrol
n	8	12	12
HR (bpm)	491±12	439±19*	463±15
E-wave (mm/s)	715±34	651±33	672±31
A-wave (mm/s)	443±17	498±18*	422±23
E/A Ratio	1.61±0.09	1.3±0.08*	1.59±0.11
IVRT (ms)	13.4±0.4	16.9±0.9*	14.1±0.3
DT (ms)	21.4±1.8	28.9±2.1*	23.5±2.4
EWDR (mm/s^2)	33.4±2.1	22.5±3.4*	28.5±2.5
E′ (mm/s)	31.8±3.2	24.3±2.6*	32.2±1.9
A′ (mm/s)	23.6±2.1	30.2±2.3*	25.6±1.7
E′ /A′	1.35±0.06	0.8±0.05*	1.26±0.05
LA Size (mm)	1.62±0.06	1.98±0.11*	1.61±0.07
E/E’(Filling pressure)	22.48±11	26.79±13	22.86±16
LVEDD (mm)	3.79±0.07	3.56±0.08	3.68±0.07
LVESD (mm)	2.54±0.06	2.35±0.07	2.41±0.06
LVFS (%)	33±1.8	34±2.2	34.5±1.9
LVEF (%)	63.1±2.2	62.9±3.3	64.1±2.1
VCFc (circ/s)	6.24±0.18	6.17±0.28	6.53±0.27
LVPWT (mm)	0.66±0.07	0.65±0.06	0.69±0.04

HR=heart rate; E=early trans-mitral filling wave; A=atrial trans-mitral filling wave; E’=early tissue Doppler velocity; A’=tissue Doppler due to atrial contraction; DT=deceleration time; IVRT=isovolumetric relaxation time; EWDR=E-wave deceleration rate; LA=left atrium; LVEDD= left ventricular end diastolic dimension; LVESD=left ventricular end systolic dimension; LVFS=left ventricular fractional shortening; LVEF=left ventricular ejection fraction; VCFc=velocity of circumferential fiber shortening; LVPWT=left ventricular posterior wall thickness; *$p<0.05$ compared with all other groups.

Supplemental Table 3. Pressure-Volume loop assessment of cardiac function in early iron-overloaded WT mice

Parameters	WT+Placebo	WT+Iron	WT+Iron+Resveratrol
n	6	8	8
HR (bpm)	554±11	424±10*	509±14
EDP (mmHg)	2.6±0.5	7.1±0.91	3.5±0.5
ESP (mmHg)	105±3.4	78.9±5.1*	93±3.8
EDV(µl)	30±1.6	27.4±2.9	30.95±3.9
ESV (µl)	5.3±1.3	3.7±1.5	6.9±1.7
SV(µl)	25.6±0.9	23.8±3.5	23±2.6
CO (ml/min)	12.9±0.33	10.6±1.2	10.8±0.9
**Systolic indexes**			
EF (%)	83.6±3.1	84.3±5.9	79±5.4
dP/dt_{max} (mmHg/s)	10120±204	9397±332	10440±525
SW (mJoules)	0.41±0.018	0.32±0.031	0.35±0.037
PRSW (mJoules/µl)	0.0134±0.02	0.0120±0.012	0.0120±0.09
dP/dt_{max}/EDV(mmHg/s/µl)	333±13	318±32	307.0±23
ESPVR (mmHg/µl)	1.97±0.5	2.1±0.3	2.08±0.3
**Diastolic indexes**			
dP/dt_{min} (mmHg/s)	9675±560	5140 ±250*	8426±207
τ (Weiss) (ms)	6.0±0.4	10.6±0.3*	6.45±0.5
τ (Glantz) (ms)	6.8±0.4	10.4±0.4*	7.8±0.4
EDPVR (mmHg/µl)	0.013±0.003	0.081±0.006*	0.017±0.002

HR=heart rate; EDP=end diastolic pressure; ESP=end systolic pressure; EDV=end diastolic volume; ESV=end systolic volume; SV=stroke volume; CO=cardiac output; EF=ejection fraction; dP/dt=rate of change in LV pressure; SW=stroke work; dP/dtmax/EDV= Starling's contractile index; Tau (τ)= LV relaxation time constant; Ees=end-systolic elastance; EDPVR= end-diastolic pressure-volume relationship; *$p<0.05$ compared with all other groups.

Supplemental Table 4. Echocardiographic assessment of cardiac function in chronically iron-overloaded male WT mice

	WT+Placebo	WT+Iron	WT+Iron+Resveratrol
n	8	12	12
HR (bpm)	493±13	397±10*	441±14#
E-wave (mm/s)	706±34	628±33	713±26
A-wave (mm/s)	438±17	375±13*	451±26
E/A Ratio	1.61±0.09	1.67±0.11	1.58±0.11
IVRT (ms)	13.8±0.4	18.2±0.9*	14.3±0.3
DT (ms)	21.9±2.1	30.1±1.8*	22.5±2.2
EWDR (mm/s^2)	32.2±2.1	20.9±2.7*	31.7±2.4
E' (mm/s)	30.4±2.5	22.7±1.6*	34±2.7
A' (mm/s)	24.3±1.9	22.8±1.4	25.8±2.1
E'/A' Ratio	1.25±0.06	1±0.05*	1.31±0.07
LA Size (mm)	1.62±0.05	2.09±0.07*	1.73±0.06
LVEDD (mm)	3.92±0.06	2.49±0.05	2.44±0.05
LVESD (mm)	2.53±0.06	2.49±0.05	2.44±0.05
LVFS (%)	35.5±1.7	35.1±2.3	37.9±1.8
LVEF (%)	64.1±2.4	63.7±3.1	66.1±2.3
VCF_C	6.31±0.15	6.37±0.23	6.42±0.24
LVPWT (mm)	0.71±0.05	0.69±0.06	0.7±0.06

see Supplemental Tables 2 and 3 for abbreviations; *p<0.05 compared with all other groups; #p<0.05 compared with the WT+Iron group.

Supplemental Table 5. Echocardiographic assessment of cardiac function in chronically iron-overloaded male HJV knockout mice

	HJVKO+Vehicle	HJVKO+Iron+Placebo	HJVKO+Iron+Resveratrol
n	8	12	12
HR (bpm)	461±15	453±11	441±14
E-wave (mm/s)	636±31	679±39	587±20
A-wave (mm/s)	363±19	526±26*	331±23
E/A Ratio	1.75±0.09	1.29±0.12*	1.77±0.11
IVRT (ms)	12.8±0.5	13.5±0.8	11.8±0.6
DT (ms)	23.4±2.3	30.4±2.2*	23.8±1.9
EWDR (mm/s^2)	27.2±2.2	22.3±2.4*	24.6±2.3
E′ (mm/s)	31.4±2.6	26±1.2	26.1±1.3
A′ (mm/s)	25±1.5	27.8±1.8	20.5±0.8
E′ /A′	1.25±0.06	0.94±0.05	1.27±0.09
LA Size (mm)	1.47±0.07	2.12±0.08*	1.53±0.07
LVEDD (mm)	3.81±0.08	3.99±0.14	3.74±0.09
LVESD (mm)	2.47±0.07	2.61±0.08	2.43±0.06
LVFS (%)	35.3±1.8	34.6±2.2	35.1±2.1
LVEF (%)	62.9±2.3	63.9±3	64.3±2.6
VCFc (circ/s)	6.39±0.14	6.44±0.21	6.52±0.24
LVPWT (mm)	0.71±0.05	0.96±0.05*	0.7±0.04
HW/TL (gm/mm)	0.075 ± 0.007	0.13 ± 0.015*	0.095 ± 0.013

see Supplemental Tables 2 and 3 for abbreviations; *p<0.05 compared with all other groups; #p<0.05 compared with other HJVKO groups.

Supplemental Table 6. Pressure-Volume loop assessment of cardiac function in chronically iron-overloaded male WT mice

Parameters	WT+Placebo	WT+Iron	WT+Iron+Resveratrol
n	6	8	8
HR (bpm)	540±2.9	398±7.5*	474.1±19
EDP (mmHg)	2.9±0.4	12±0.9*	7.1±0.4
ESP (mmHg)	88±1.3	96±1.5*	87±2.9
EDV(µl)	25±1.0	26.7±2.0	25.7±1.3
ESV (µl)	6.9±0.8	6.6±0.8	7.2±0.9
SV(µl)	17.9±0.4	18.1±1.8	18.08±1.2
CO (ml/min)	9.8±0.3	7.7±0.7*	9.4±0.9
**Systolic indexes**			
EF (%)	73.9±2.2	70.1±2.6	71.9±3.7
dP/dt_{max} (mmHg/s)	8849±110	8633±172	8373±301
SW (mJoules)	0.27±0.009	0.26±0.021	0.27±0.03
PRSW (mJoules/µl)	0.011±0.008	0.010±0.11	0.011±0.023
dP/dt_{max}/EDV(mmHg/s/µl)	342±22	340±36	363±31
ESPVR (mmHg/µl)	3.3±0.6	3.6±0.5	3.6±0.6
**Diastolic indexes**			
dP/dt_{min} (mmHg/s)	7900±309	6693±83.17*	7415±363.7
τ (Weiss) (ms)	6.2±0.095	7.7±0.19*	6.7±0.39
τ (Glantz) (ms)	8.8±0.36	11.21±0.38*	8.5±0.42
EDPVR (mmHg/µl)	0.021±0.008	0.082±0.019*	0.039± 0.013

see Supplemental Tables 2 and 3 for abbreviations; *p<0.05 compared with all other groups.

Supplemental Table 7. Pressure-Volume loop assessment of cardiac function in chronically iron-overloaded male HJV knockout mice

Parameters	HJVKO+Vehicle	HJVKO+Iron+Placebo	HJVKO+Iron+Resveratrol
n	6	8	8
HR (bpm)	431±8	426±9	443±14
EDP (mmHg)	9.86±1.43	16.2±1.95*	8.86±1.53
ESP (mmHg)	103±6.1	106.7±7.4	110.3±6.3
EDV(µl)	22.5±2.8	24.7±2.6	23.7±2.2
ESV (µl)	4.36±1.72	4.7±1.38	4.12±0.72
SV(µl)	18.2±1.7	20±1.9	19.9±2.1
CO (ml/min)	8.67±0.22	8.52±0.26	8.84±0.23
**Systolic indexes**			
EF (%)	79.1±3.8	80.4±5.3	83.7±5.1
dP/dt_{max} (mmHg/s)	9637±135	9201±149	9781±172
SW (m Joules)	0.341±0.062	0.382±0.049	0.393±0.056
PRSW (m Joules/µl)	0.016±0.023	0.016±0.019	0.017±0.026
dP/dt_{max}/EDV (mmHg/s/µl)	428±31	372±45	413±37
ESPVR (mmHg/µl)	3.14±0.35	3.08±0.29	3.75±0.32
**Diastolic indexes**			
dP/dt_{min} (mmHg/s)	8003±177	8228±156	8740±303
τ (Weiss) (ms)	7.11±0.65	9.72±0.41*	7.22±0.46
τ (Glantz) (ms)	9.23±0.41	13.7±0.32*	9.49±0.49
EDPVR (mmHg/µl)	0.017±0.006	0.112±0.013*	0.014±0.005

see Supplemental Tables 2 and 3 for abbreviations; *p<0.05 compared with all other groups.

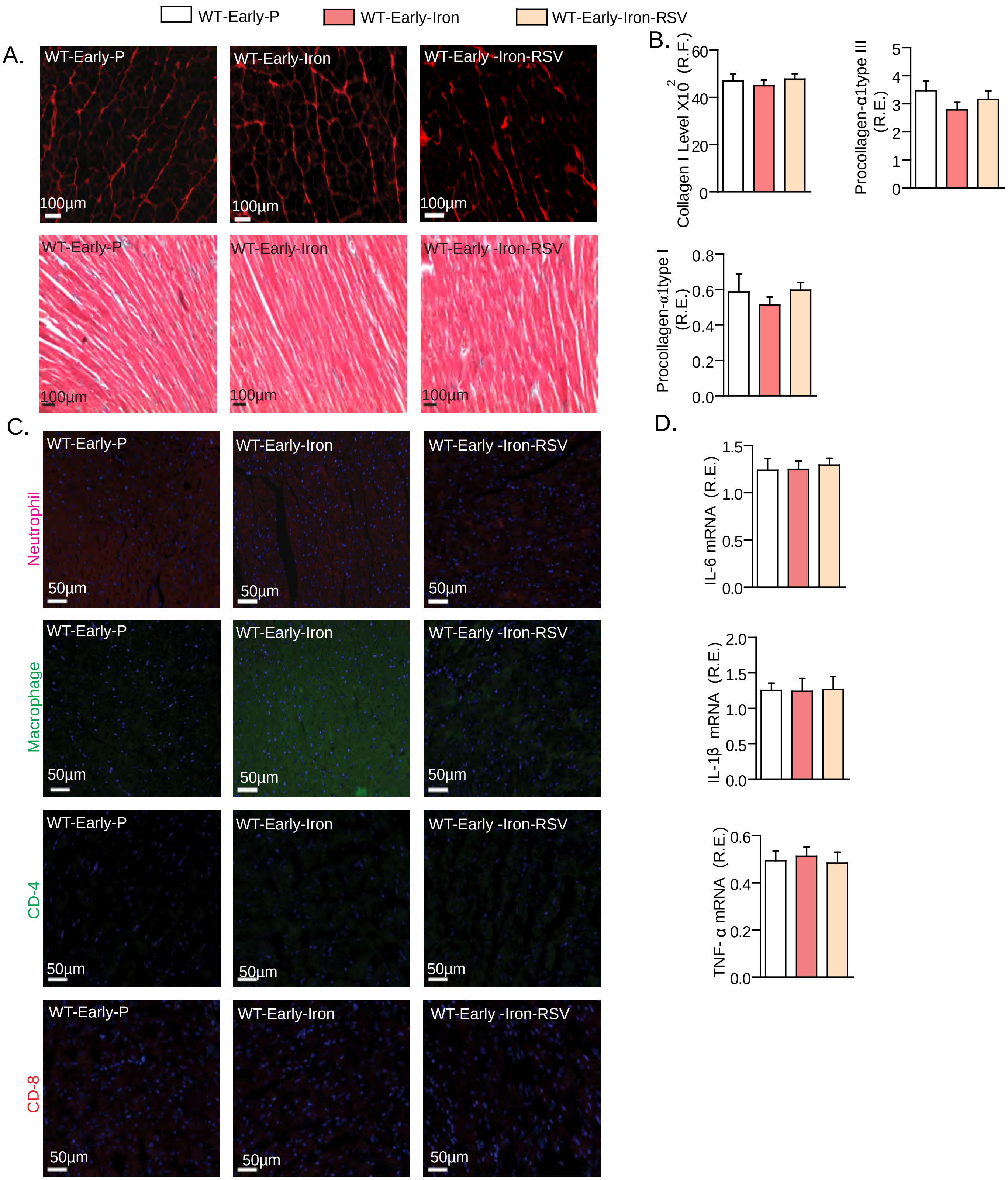

Supplemental Figure 1. Picrosirius red (top panel) and Trichrome (bottom panel) staining (**A**), and collagen quantification and expression analysis of pro-collagen Iα1 and pro-collagen IIIα1 (**B**) showing lack of myocardial fibrosis in early iron-overloaded wildtype hearts. Immunohistochemical staining for neutrophil, macrophages, CD4 and CD8 positive lymphocytes (**C**) and gene expression analysis of pro-inflammatory cytokines (**D**) showing lack of myocardial inflammation in early iron-overloaded wildtype hearts. IL-6=interleukin-6; IL-1β=interleukin-1 beta; TNFα=tumor necrosis factor alpha. n=6 for the placebo and n=8 for the iron-overloaded groups

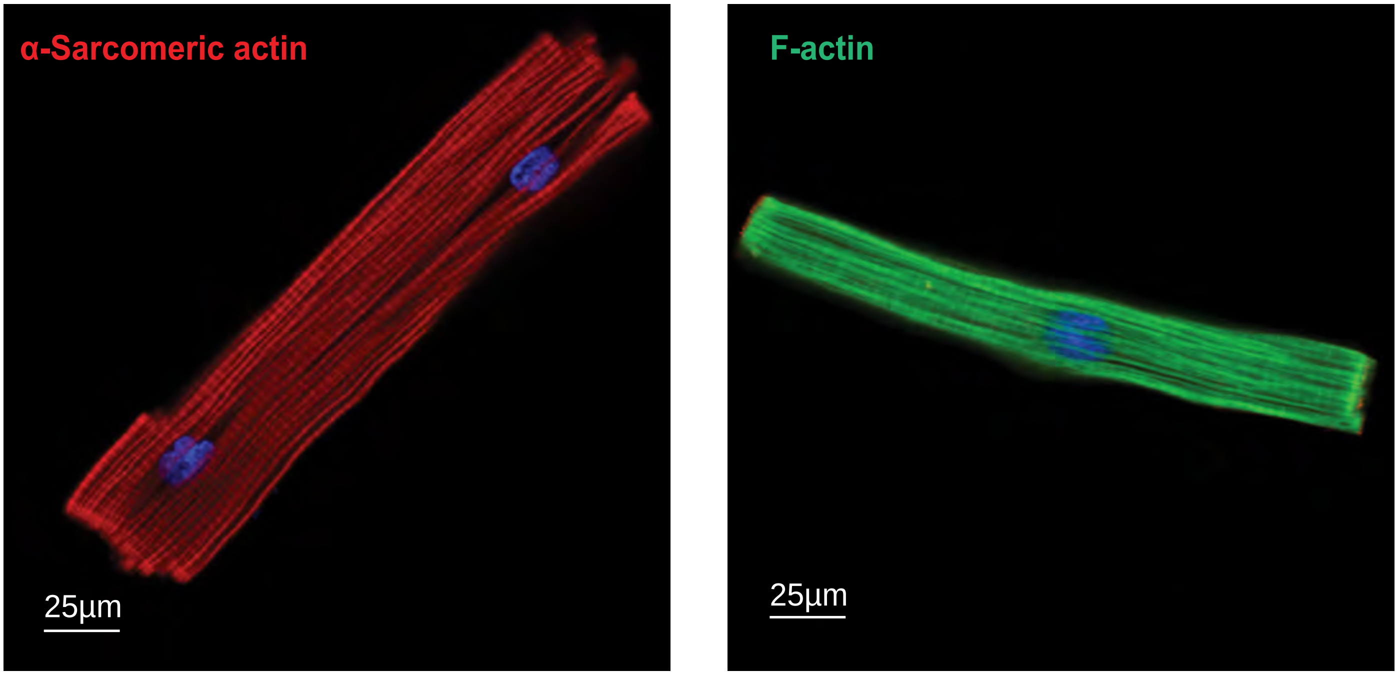

Supplemental Figure 2. Human cardiomyocytes isolated from the left ventricle of a non-failing healthy control heart display a normal morphology illustrated by alpha-sarcomeric actin staining (red) and F-actin staining (green).

Das SK. *et al.* Supplemental Figure 2

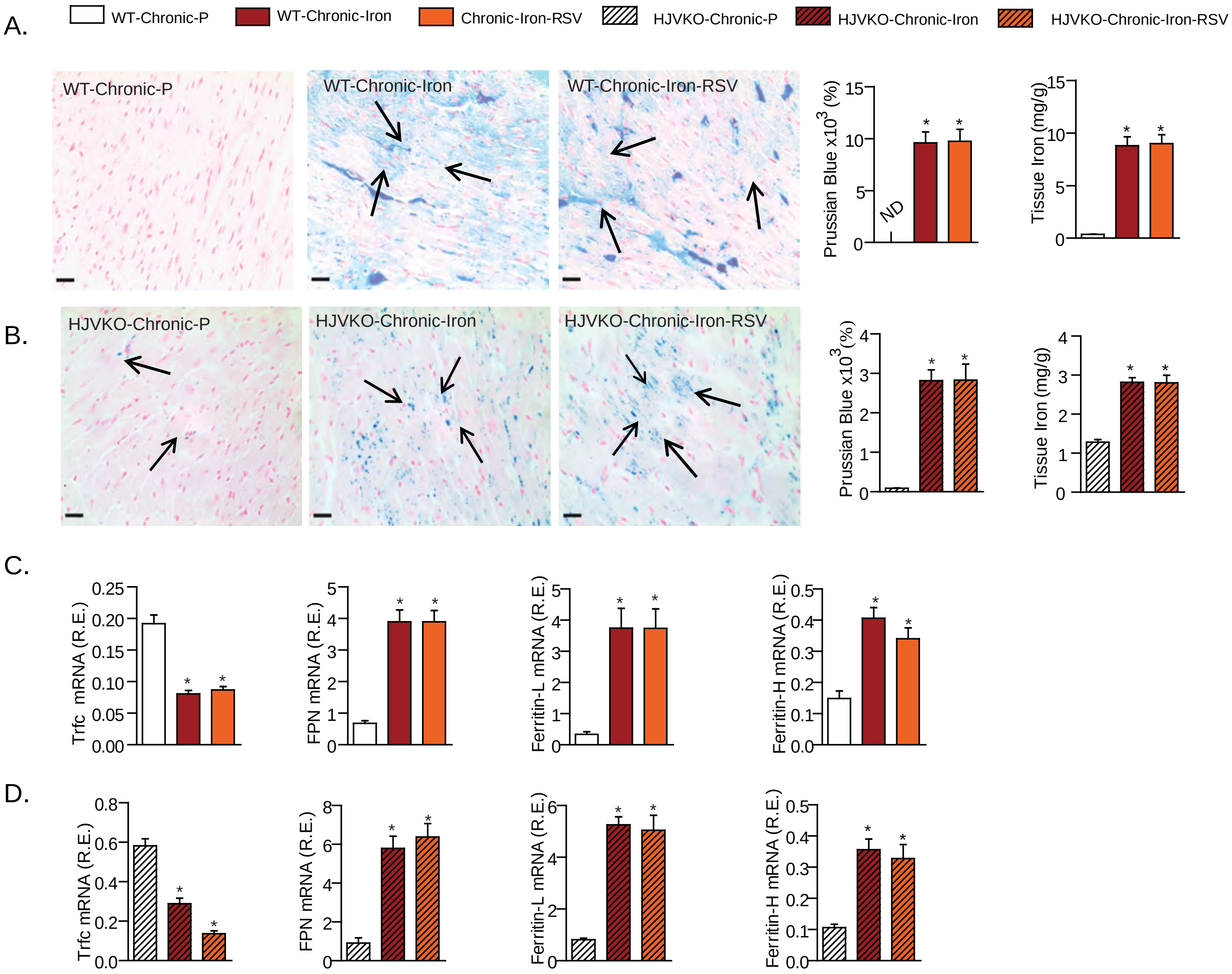

Supplemental Figure 3. Prussian blue staining with quantification of myocardial iron levels in the hearts of chronically iron-overloaded wildtype (**A**) and hemojuvelin knockout (HJVKO) mice (**B**) showing myocardial iron-overload with resveratrol (RSV) having no effect on the degree of myocardial iron-overload. Myocardial gene expression analysis revealed that the heart was clearly sensitize to chronic iron injury as reflected in the altered expression of iron metabolic genes, transferrin receptor 1 (Trfc), ferroportin (FPN), and ferritin light (L) and heavy (H) chain in chronically iron-overloaded wildtype (**C**) and HJVKO mice (**D**). Resveratrol did not alter the expression pattern of these iron metabolism genes. n=6 for placebo and n=8 for iron-treated groups. ND=not detected; *p<0.05 compared with the placebo group

Das SK. *et al.* Supplemental Figure 3

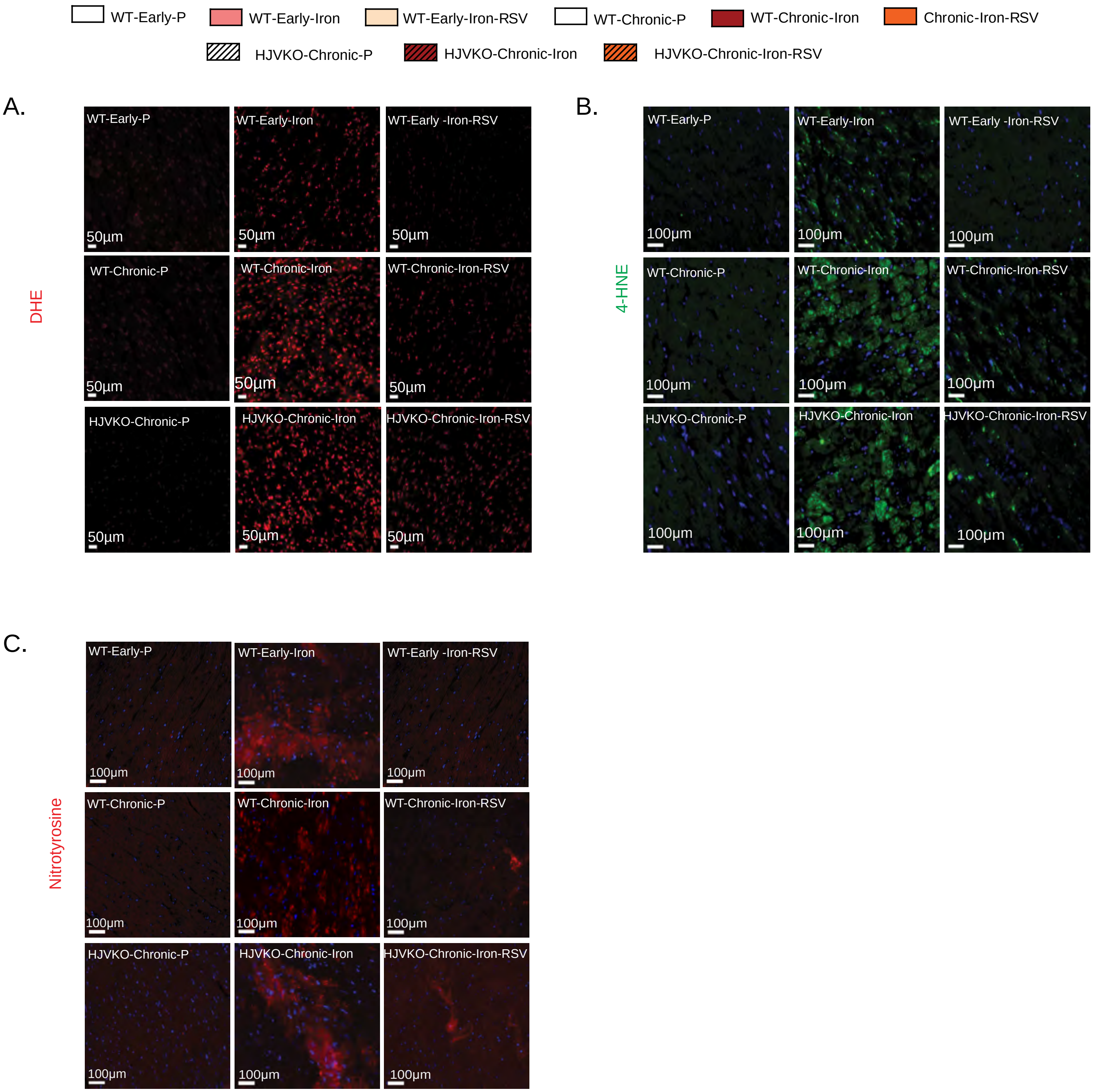

Supplemental Figure 4. Resveratrol has potent antioxidant properties in iron-overloaded hearts based on dihydroethium (DHE) staining for superoxide levels (**A**), 4-hydroxynonenal (4-HNE) immunofluorescence (**B**), nitrotyrosine (NT) immunofluorescence (**C**), showing marked increases in iron-induced oxidative stress in early and chronic iron-overloaded hearts with a drastic reduction in response to dietary resveratrol. Myocardial oxidative stress was minimal in placebo-treated mice.

Das SK. *et al.* Supplemental Figure 4

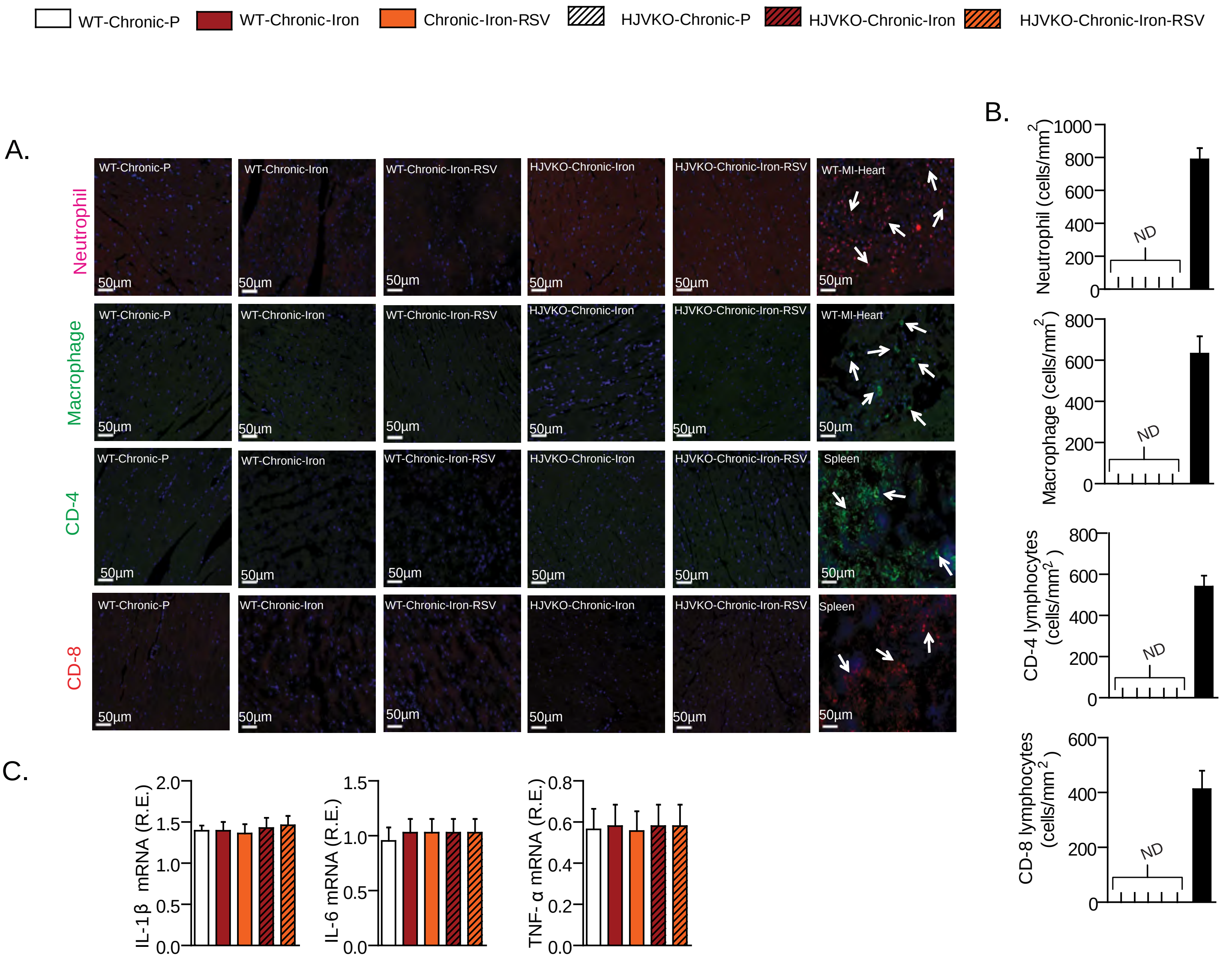

Supplemental Figure 5. Lack of myocardial inflammation in chronic iron-overloaded mice based on immunohistochemical staining for neutrophils, macrophages, CD4 and CD8 positive lymphocytes (**A**) and quantification of inflammatory cells using positive controls, 3-day post-myocardial infarction hearts and spleen (**B**). Inflammatory cells are highlighted with white arrows. Gene expression analysis of pro-inflammatory cytokines confirmed a lack of myocardial inflammation in response to chronic iron-overload (**C**). ND=not detected; IL-6=interleukin-6; IL-1β=interleukin-1 beta; TNFα=tumor necrosis factor alpha.

Das SK. *et al.* Supplemental Figure 5
